# Adaptive Flight Maneuver Boundary Localization via Spectral Entropy-Weighted Multi-Channel Spectrogram Fusion

**DOI:** 10.3390/e28080922

**Published:** 2026-08-17

**Authors:** Shansong Song, Wei Han, Bing Wan, Xiangyi Liu, Xichao Su, Chao Li, Yunyang Cao

**Affiliations:** 1 School of Aeronautical Fundamentals, Naval Aviation University, Yantai 264001, China; 2 Coastal Defense Academy, Naval Aviation University, Yantai 264001, China; 3 School of Aviation Operation and Support, Naval Aviation University, Yantai 264001, China; 4 Unit 92913 of PLA, Lingao 571800, China

**Keywords:** multi-channel spectrogram fusion, boundary localization, spectral entropy, Short-Time Fourier Transform, boundary detection

## Abstract

To address ambiguous maneuver boundaries, background interference, and uneven multi-sensor quality in long-duration flight parameter recordings, this paper proposes an adaptive flight maneuver boundary localization algorithm that integrates spectral entropy-weighted multi-channel spectrogram fusion with attitude-constrained structural correction. Multi-channel Short-Time Fourier Transform (STFT) spectrograms are first constructed from flight parameter time series. Spectral entropy (SE) is introduced to quantify the uncertainty of each channel’s time–frequency energy distribution and is combined with the maneuver activation ratio (MAR) and the linear contrast ratio (LCR) to form objective credibility weights, thereby suppressing channels dominated by aerodynamic turbulence and high frequency structural vibration. Normal overload soft gating and logarithmic noise floor subtraction are then applied to obtain an enhanced fused spectrogram, from which candidate intervals are extracted by low band energy thresholding. Finally, roll and pitch angle steady-state priors refine the event structure through local boundary refinement, cross-segment expansion/chain merging, and semantic post-processing, recovering continuous maneuvers fragmented by instantaneous energy valleys. On the held-out test sorties (SE_018–SE_020; 61 annotated intervals), the proposed algorithm achieves Precision, Recall, and F1-scores of 0.967. On the full primary corpus of 20 sorties (461 intervals), used for ablation and sensitivity analyses, the corresponding figures are Precision 0.934, Recall 0.959, and F1 0.946, with start and end boundary mean absolute errors of 1.484 s and 1.471 s. Under the same IoU protocol, consistent superiority is observed against learning-based baselines, and an independent external set of 10 sorties yields F1 = 0.938. The results indicate that entropy-constrained multi-sensor time–frequency fusion mainly improves maneuver/background separability, whereas attitude-constrained structural correction restores the integrity of long continuous maneuvers.

## 1. Introduction

Precisely segmenting flight maneuvers from multi-channel flight parameter records is a critical prerequisite for pilot training evaluation and aircraft fatigue estimation, where boundary localization precision directly dictates the fidelity of downstream metrics [1,2,3]. Errors of even a few seconds at maneuver onset or termination propagate into maneuver recognition, competency scoring, and structural load spectrum analysis. However, current automated methods still rely primarily on time domain amplitude thresholds that struggle to achieve temporally precise boundary localization, because flight maneuvers are strongly nonlinear and tightly coupled across channels, and their onset and termination signatures are easily obscured by aerodynamic turbulence and high frequency structural vibration that single-channel time domain criteria fundamentally fail to resolve. Developing adaptive multi-channel spectrogram fusion and precise boundary correction methods is therefore essential to overcome this bottleneck, providing critical support for objective pilot competency grading and reliable aircraft structural health diagnostics.

Flight maneuver recognition and segmentation methods fall into three categories: rule-based and expert systems, feature engineering coupled with probabilistic modeling, and data-driven neural networks [4]. Rule-based and expert knowledge systems represent the pioneering approach. Wang et al. [5] quantified key maneuver parameters—normal overload, attitude angles, and angular rates—into a structured feature database, enabling automatic identification and temporal annotation through pattern matching. Yang et al. [6] incorporated fuzzy membership functions to construct expert threshold rules covering boundary transition zones, while Zhang et al. [7] developed a normal overload trend-clustering procedure that reduces reliance on manual feature engineering, reporting a correct recognition rate of no less than 89% on large-scale flight data. Wang et al. [8] further integrated multi-source airborne parameters for civil aviation flight phase partitioning. These methods offer clear physical interpretability and low computational cost, but boundary localization reduces to fixed threshold crossings on individual channels that drift systematically under turbulence; no correction mechanism compensates for this boundary bias, and rule bases are tightly coupled to specific aircraft types, limiting cross-platform generalization [9].

Feature engineering and probabilistic methods improved generalization to varying degrees. Zhang et al. [10] proposed an improved DTW algorithm with an adaptive constraint band, reducing temporal alignment distortion from execution speed variation across maneuver instances. Meng et al. [11] applied dynamic Bayesian networks for structured probabilistic inference with compact parameter sets. Olive and Basora [12] implemented a multivariate HMM that performs boundary detection through latent-state transition modeling across six flight phases over several thousand flights, competitive with fuzzy logic baselines; this is the only study in this category to produce boundary timestamps, yet the state granularity is restricted to the coarse flight phase level, and temporally precise boundary localization was not evaluated. Lu et al. [9] segmented flights via unsupervised trend clustering and then classified each segment by its sequence complexity using phase-space reconstruction and approximate entropy [13], reaching 85.5% average recognition accuracy across three aircraft types without retraining; yet because segments are distinguished by whole-sequence complexity, their boundaries are only a by-product of clustering rather than explicitly modeled, so the method cannot localize maneuver onset and termination with the required temporal precision. Collectively, these methods improve generalization over rule-based approaches, but none provides the temporally precise boundary localization required for individual maneuver analysis.

Data-driven deep learning has brought the most substantial gains in recognition capability while introducing new constraints. Tian et al. [14] combined a deep variational autoencoder with LSTM networks and adaptive DTW to extract latent maneuver dynamics in an unsupervised manner, enabling concurrent quality evaluation without dense boundary labels, though it operates on pre-segmented intervals and does not address boundary localization. Wang et al. [15] formulated recognition as temporal detection through FM-detector—a feature extraction backbone, a region proposal network for boundary candidate generation, and a classification–regression head—reporting over 97% mAP with higher-quality predicted boundaries than prior learning-based models; this is the only aviation deep-learning study that explicitly predicts both onset and termination, though boundary precision is constrained by fixed anchor scales that degrade for maneuvers deviating from preset duration templates. Fang et al. [16] and Zhou et al. [17] improved recognition through symbolic sequence modeling and multivariate channel fusion, respectively, while Li et al. [18] validated end-to-end deep pipelines for complex military maneuver extraction. Xi et al. [19] addressed air combat scenarios through a combined autoencoder and time series segmentation approach that extracts maneuver primitives without empirical criteria, though it operates on trajectory-level features rather than raw multi-channel parameters and does not evaluate boundary timestamp accuracy.

Two model families are widely regarded as the current state of the art for temporal segmentation, and each exposes a specific limitation for precise maneuver boundary localization. The first is attention- and Transformer-based temporal modeling. Within flight regime segmentation, Wu et al. [20] proposed DTS-Net, a multi-scale attention network performing point-wise prediction over long sequences without pre- or post-processing, which attains 95.98% recognition accuracy across 11 flight regimes yet explicitly acknowledges that the resulting boundaries remain imprecise owing to absent boundary supervision; Hu et al. [21] recast the task as temporal detection using adaptive graph convolution and multi-scale Transformer encoding to achieve boundary-sensitive regime recognition, but at coarse flight phase granularity and with dependence on large frame-level annotated corpora. Beyond aviation, Gaugel and Reichert [22] proposed PrecTime, a CNN–BiLSTM sequence-to-sequence architecture with a dedicated refinement stage that raises change point precision and recall to 59.5% and 75.6% at roughly 96% segmentation accuracy on industrial multivariate sensor data, and Bock et al. [23] showed that Temporal Action Localization models improve boundary coherence by treating the whole timeline as context, yielding F1 gains of up to 26% over sliding window approaches across human activity recognition benchmarks. The second family is self-supervised and representation learning, which seeks to reduce annotation dependence: the VAE–LSTM scheme [14] and the autoencoder-based primitive extraction [19] already learn latent maneuver dynamics without dense labels, and more general contrastive or masked reconstruction pretraining on unlabeled multivariate time series lowers the downstream labeling burden. Both families, however, share two obstacles that leave the present problem unsolved: they require large annotated (Transformer detection) or large unlabeled (self-supervised) corpora to reach stable performance, and their learned representations are not physically interpretable and do not directly emit calibrated onset/termination timestamps—precise boundaries must still be decoded by a separate stage, and reported evaluations remain at coarse flight phase rather than individual maneuver granularity.

A comparative overview is provided in Table 1. Although several learning-based methods report strong recognition scores, these are classification-oriented metrics obtained on heterogeneous datasets and protocols; crucially, high frame-level accuracy does not imply temporally precise boundaries—DTS-Net, for example, reports high recognition accuracy while explicitly acknowledging that its boundaries remain imprecise. A consistent gap therefore remains: no existing method simultaneously exploits complementary multi-sensor information while suppressing low quality channels, achieves temporally precise maneuver boundary localization robust to broadband vibration backgrounds, and preserves physical interpretability without large-scale labeled training. Recognition accuracy and precise boundary localization are thus distinct objectives, and it is the latter—largely unaddressed in the small-sample, label-scarce setting of flight testing—that this work targets.

Addressing this gap requires a shift from time domain amplitude thresholds toward a frequency domain framework. During maneuver execution, control inputs and aerodynamic responses cause sustained energy concentration in the low frequency region of angular rate, overload, and attitude channels, whereas steady cruise, ground taxiing, and turbulence backgrounds manifest as weak responses or broadband dispersion, making the spectrogram domain a natural discriminative space for boundary localization. The STFT is the most mature tool for characterizing the local spectral evolution of non-stationary signals [24], and spectral entropy has been shown to outperform pure energy criteria for endpoint detection at low signal-to-noise ratios [25,26], to improve boundary delineation in heterogeneous sensor segmentation [27], and to weight source uncertainty in multi-sensor fusion [28,29]. Three domain-specific obstacles must nevertheless be overcome: heterogeneous channel sensitivity across maneuver types, a broadband vibration floor from taxiing and engine operation requiring frequency-dependent noise subtraction [30], and the systematic inward boundary bias introduced by STFT windowing under non-stationary noise.

An adaptive, unsupervised segmentation method is therefore proposed that combines spectral entropy-weighted multi-channel spectrogram fusion with attitude-constrained structural correction. Spectrograms of the three-axis angular rates and the normal and lateral overloads are fused using credibility weights built jointly from spectral entropy, the Maneuver Activation Ratio (MAR), and the Linear Contrast Ratio (LCR), then enhanced by soft gating and frequency-wise noise floor subtraction; candidate intervals are refined under roll and pitch steady-state priors through local boundary fine tuning, cross-segment expansion, and co-directional semantic merging. The main contributions are: (1) an adaptive multi-channel spectrogram fusion framework in which a joint MAR–LCR–SE channel quality metric weights sensor channels by their maneuver response credibility, with soft gating and frequency-wise noise floor subtraction further enhancing the fused spectrogram against ground vibration and steady flight backgrounds; (2) a temporally precise boundary localization method that introduces roll and pitch steady-state priors into a three-step structural correction procedure of local boundary fine tuning, cross-segment expansion, and co-directional semantic merging, reducing the boundary mean absolute error to approximately 1.5 s; (3) an interpretable, fully unsupervised, and training-free two-stage framework that decouples multi-channel fusion from attitude-constrained boundary correction, requires no labeled training corpus, and therefore remains applicable to the small-sample datasets typical of flight testing, with component-wise effectiveness verified through staged ablation on real flight data and generalization confirmed on an independent external set.

The remainder of this paper is organized as follows: Section 2 analyzes flight maneuver signal characteristics; Section 3 details STFT spectrogram construction and parameter optimization; Section 4 presents channel quality evaluation and multi-channel fusion enhancement; Section 5 describes candidate detection and attitude-constrained boundary correction; Section 6 reports comparative and ablation experiments; and Section 7 concludes.

## 2. Signal Characteristics of Flight Maneuvers

### 2.1. Non-Stationary Characteristics of Flight Maneuvers

**Figure 1:**
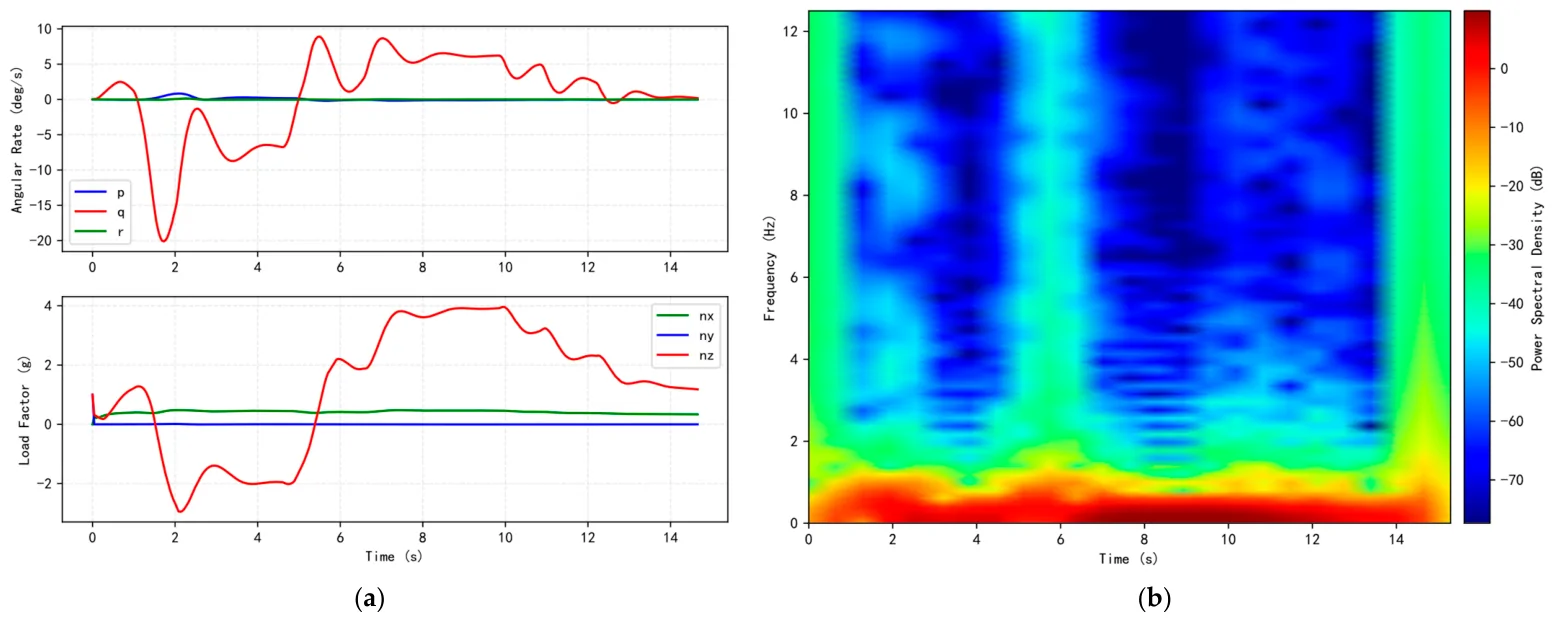
Analysis of flight parameter characteristics during a dive maneuver. (**a**) Time series of key flight parameters. (**b**) Time frequency energy distribution of $n_{z}$.

A flight maneuver is a dynamic process in which the pilot manipulates the control stick, rudder, and other mechanisms, causing complex nonlinear evolution of aircraft attitude, trajectory, and aerodynamic loads. From a signal-processing perspective, this time-varying coupling among multiple parameters constitutes a typical non-stationary stochastic process. During steady level flight, the statistical characteristics of flight parameter sequences are relatively stable; during maneuver entry and recovery, however, signal energy migrates sharply in the time frequency plane. Taking a dive maneuver as an example, Figure 1a shows the time series of key flight parameters, in which the pitch rate q and normal overload nz exhibit strong amplitude fluctuations within the maneuver interval, reflecting the maneuver’s excitation of aircraft dynamics. Figure 1b presents the corresponding time frequency evolution: during maneuver entry and recovery, low frequency signal energy exhibits pronounced instantaneous changes. This non-stationary evolution of energy distribution not only characterizes the maneuver dynamics directly, but also provides an effective feature criterion for determining maneuver boundaries, thereby supporting automatic segmentation and recognition based on time frequency analysis.

Although this energy evolution pattern reveals the dynamic nature of maneuvers, converting it into a general boundary criterion remains challenging in engineering practice. First, pilot-specific operation habits lead to variable maneuver durations, and adjacent maneuvers often contain ambiguous transition segments. Second, conventional global spectral analysis is unable to accurately localize abrupt frequency component changes because its basis functions extend infinitely in time. Direct fixed window segmentation can also introduce truncation errors and information distortion at sharp signal transitions. Therefore, to overcome the recognition difficulties caused by non-stationarity, a mathematical representation capable of simultaneously describing local temporal characteristics and instantaneous frequency evolution is required for reliable maneuver segmentation.

### 2.2. Construction of the Discriminative Parameter Space for Flight Maneuvers

A typical flight data recording system contains hundreds of parameter channels, but not all parameters contribute equally to maneuver recognition. Considering the noise and computational overhead introduced by redundant parameters, and following flight dynamics principles and prior research experience [1,28], this study selects the following seven core parameters that best characterize the dynamic response of aircraft spatial attitude and loading to construct the flight-state observation vector x(t):

(1) $$x(t)={\left[p(t),q(t),r(t),n_{y}(t),n_{z}(t),\phi (t),\theta (t)\right]}^{T}$$

where $p\left(t\right),q\left(t\right),r\left(t\right)$ denote the three-axis angular rates (unit: deg/s), directly reflecting the instantaneous rotational state of the aircraft body; $n_{y}\left(t\right)$ and $n_{z}\left(t\right)$ denote the lateral and normal overloads (unit: g), characterizing the aerodynamic load response during maneuver execution; and $\phi \left(t\right)$ and $\theta \left(t\right)$ denote the roll angle and pitch angle (unit: degrees), representing the spatial attitude evolution. These seven features offer strong discrimination for maneuvers like dives, rolls, and turns. Specifically, five dynamic channels feed the STFT fusion, whereas the attitude angles (phi, theta) are used solely for trend-aware boundary correction.

The objective of automated maneuver segmentation is to automatically extract *K* independent maneuver segments with significant dynamic significance along the time axis. The set of maneuver segments is defined as

(2) $$A=\left\{\left.\left(t_{s}^{i},t_{e}^{i}\right)\right|1\leq i\leq K,t_{s}^{i}<t_{e}^{i}\right\}$$

where $t_{s}^{i}$ and $t_{e}^{i}$ are the start and end times of the *i*-th maneuver segment, respectively. Given the boundary ambiguity of certain maneuvers, this work uses roll angle ϕ and pitch angle θ as fine-tuning factors on top of the initial energy-based segmentation to improve boundary localization accuracy.

## 3. STFT Spectrogram Construction and Parameter Optimization

### 3.1. Short-Time Fourier Transform and Spectrograms

To characterize the instantaneous frequency properties of the non-stationary flight maneuver signal and map the one-dimensional time series to the two-dimensional time frequency plane, the Short-Time Fourier Transform (STFT) is applied. Before performing the STFT, the signal is preprocessed using linear interpolation and forward/backward filling, omitting high-pass detrending to preserve the physical 1 g $n_{z}$ bias. This bias is subsequently suppressed via soft gating and frequency-wise log spectrogram noise floor subtraction. Let the preprocessed discrete time sequence of the *i*-th flight parameter channel be $x_{i}\left(n\right)$ at sampling frequency $f_{s}$. Its discrete STFT is defined as

(3) $$X_{i}\left(m,k\right)=\overset{L-1}{\underset{n=0}{\sum }}x_{i}\left(n+mH\right)w\left(n\right)e^{-j\frac{2\pi }{N}kn}$$

where *m* is the frame index, *k* is the discrete frequency bin index, *H* is the hop size, *N* is the Discrete Fourier Transform (DFT) length, and $w\left(n\right)$ is the analysis window function of length *L* (e.g., a Hann window), with n=0,1,…,L−1 denoting the local time index relative to the start of the current frame. Through the truncation effect of the window function, the STFT achieves local stationarity within short time windows, thereby transforming a globally long duration signal into a series of frequency distributions evolving over time.

To quantitatively characterize the energy strength of each frequency component within a specific time window, the magnitude of the STFT output is squared to construct the spectrogram (power spectral density):

(4) $$S_{i}\left(m,k\right)={\left|X_{i}\left(m,k\right)\right|}^{2}$$

To further compress the dynamic range and enhance weak features, this is commonly converted to a logarithmic scale (in dB): $10\log_{10}\left(S_{i}\left(m,k\right)\right)$. The resulting spectrogram (as illustrated in Figure 1b) has time frames on the horizontal axis and frequency on the vertical axis, intuitively revealing the temporal evolution of the maneuver signal’s energy.

**Figure 2:**
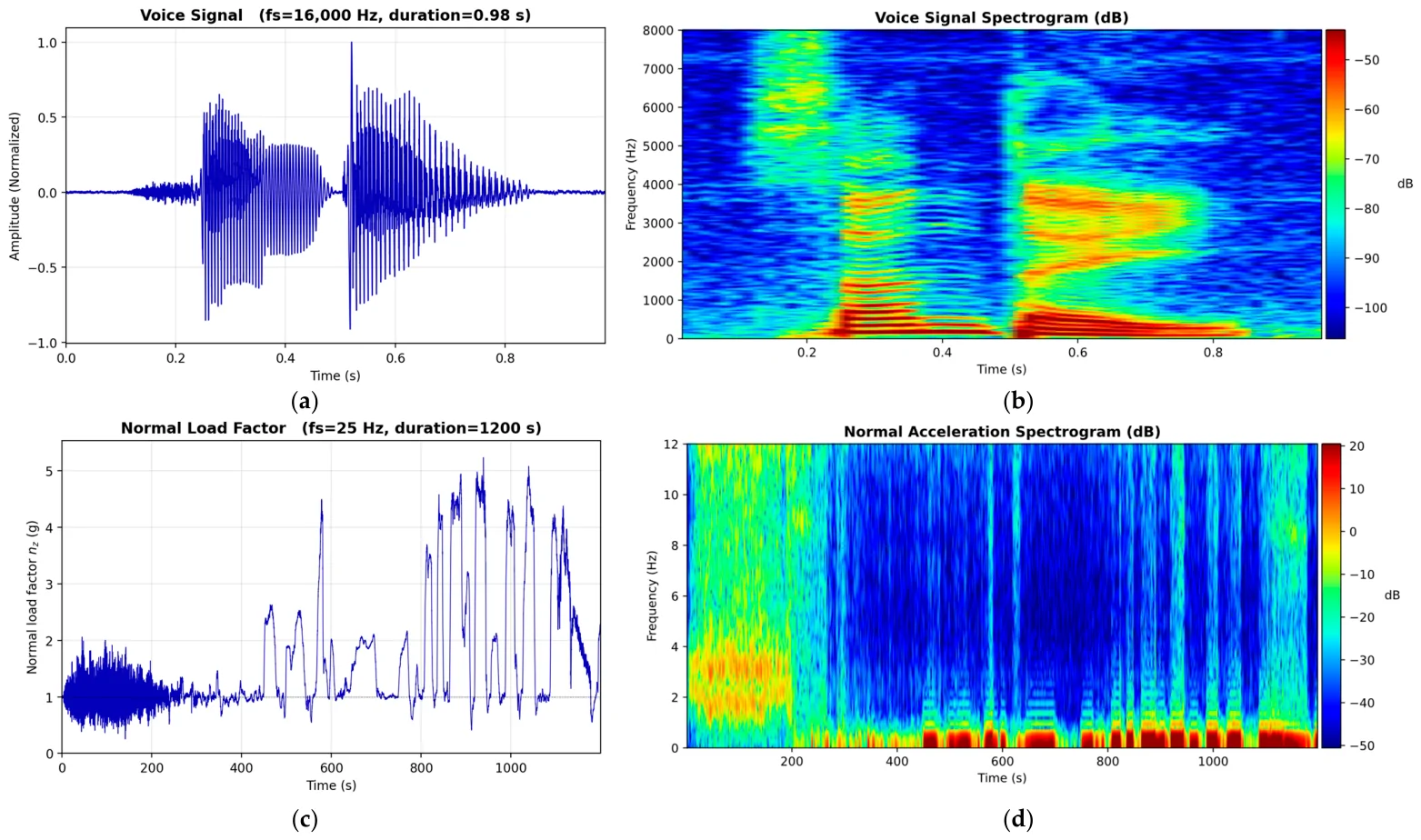
Comparison of waveforms and spectrograms between a speech signal and a flight parameter signal. (**a**) Time domain waveform of speech signal; (**b**) spectrogram of speech signal; (**c**) time domain waveform of normal acceleration; (**d**) spectrogram of normal acceleration.

To justify the application of spectrogram analysis to flight parameters, a comparative study was conducted between a representative speech signal and a flight normal acceleration signal, with the comparison of their spectrogram analyses shown in Figure 2.

As shown in Figure 2, despite the significant disparity in sampling frequencies, where the speech signal is sampled at 16 kHz compared to the 25 Hz rate of the flight data, both signals exhibit homologous time frequency characteristics characterized by localized energy concentration and dominant frequency band evolution. The primary energy of the flight data resides within the low frequency region below 2 Hz, where maneuver segments manifest as continuous or impulsive high energy textures within the range of several hertz. In contrast, steady-state flight and ground operation phases present relatively dispersed time frequency responses. This phenomenon indicates that flight maneuvers are not merely characterized by singular amplitude exceedances, but embody local time frequency features effectively revealed by the Short-Time Fourier Transform. Although the resonant peaks and harmonic structures of speech signals occur at the kilohertz level and contrast sharply with the low frequency nature of maneuver responses, appropriate adjustments to the short-term window parameters allow for the effective capture of dynamic processes in both signals. Consequently, the high energy textures in the flight spectrogram, which correlate closely with abrupt changes in normal acceleration, demonstrate that spectrograms can integrate multidimensional information such as energy intensity, duration, and frequency distribution, thereby providing a robust empirical basis for the subsequent development of an energy fusion-based maneuver segmentation model.

### 3.2. Window Function Selection and Parameter Optimization

In STFT, frame truncation makes the signal discontinuous at frame endpoints, and direct DFT therefore produces severe spectral leakage. For flight maneuver signals, whose energy is mainly concentrated in the low frequency band of 0–2 Hz, severe spectral leakage causes maneuver-induced low frequency energy to spread into high frequency noise regions, blurring the energy contrast between maneuver and stationary segments in the time frequency map and reducing subsequent segmentation reliability. Windowing smooths the framed signal toward zero and effectively suppresses leakage.

**Figure 3:**
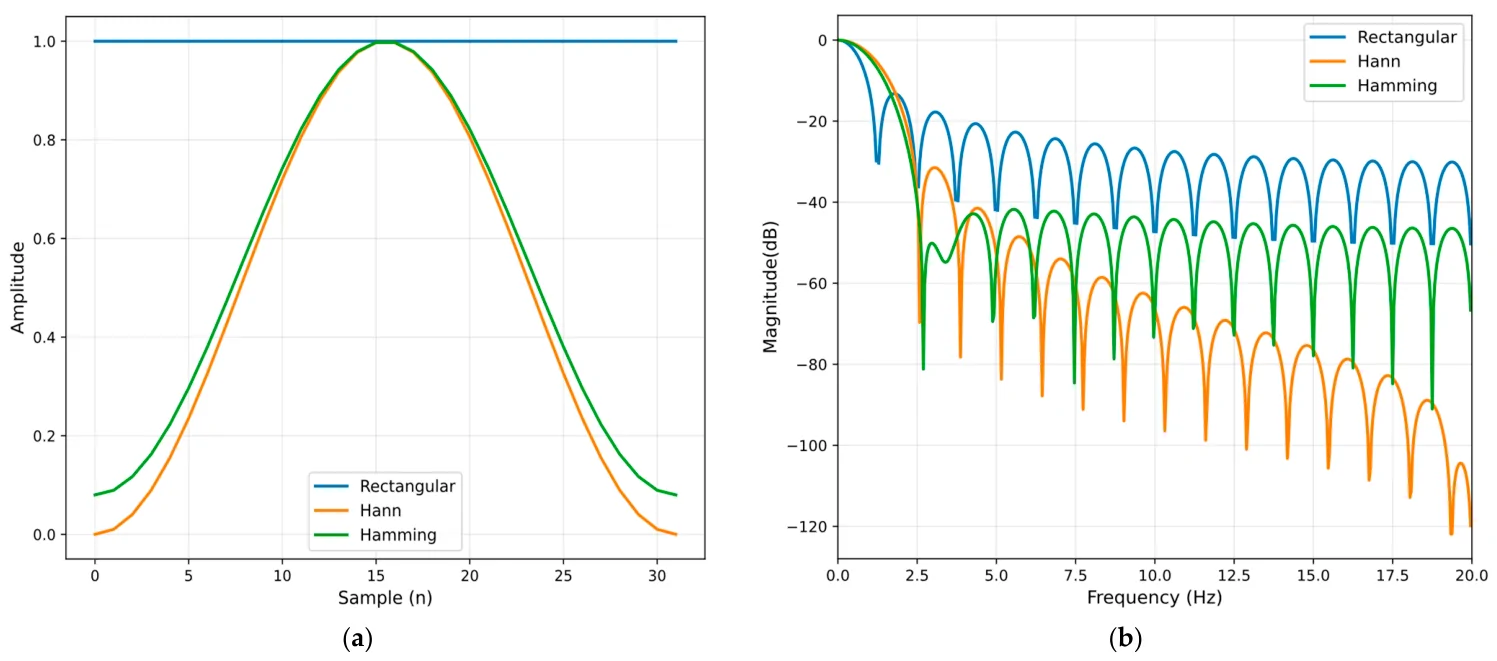
Time domain waveforms and normalized frequency domain magnitude response curves of common window functions. (**a**) Time domain waveforms; (**b**) normalized frequency domain magnitude responses.

The commonly used window functions considered here are the rectangular, Hamming, and Hann windows. Their time domain waveforms and normalized frequency domain magnitude responses are shown in Figure 3.

As shown in Figure 3a, the rectangular window assigns uniform weights to all samples within a frame and exhibits discontinuous step changes at the start and end boundaries. The Hamming window introduces cosine modulation to smooth the edges, but its endpoint value is approximately 0.08 and does not strictly reach zero. The Hann window is exactly zero at both ends and provides the smoothest boundary transition. Abrupt amplitude changes in the time domain correspond to pronounced spectral leakage in the frequency domain. Figure 3b shows that the rectangular window has the slowest sidelobe decay and the strongest leakage, the Hamming window improves the first sidelobe peak but retains appreciable high frequency energy owing to its non-zero endpoints, and the Hann window decays most rapidly with the deepest high frequency suppression. Under the adopted STFT setting (*L* = 64, $f_{s}$ = 25 Hz), Table 2 gives mean sidelobe levels above 4 Hz of −47.5, −57.3, and −97.2 dB for the rectangular, Hamming, and Hann windows, respectively; the Hann window is therefore selected for spectrogram construction.

**Figure 4:**
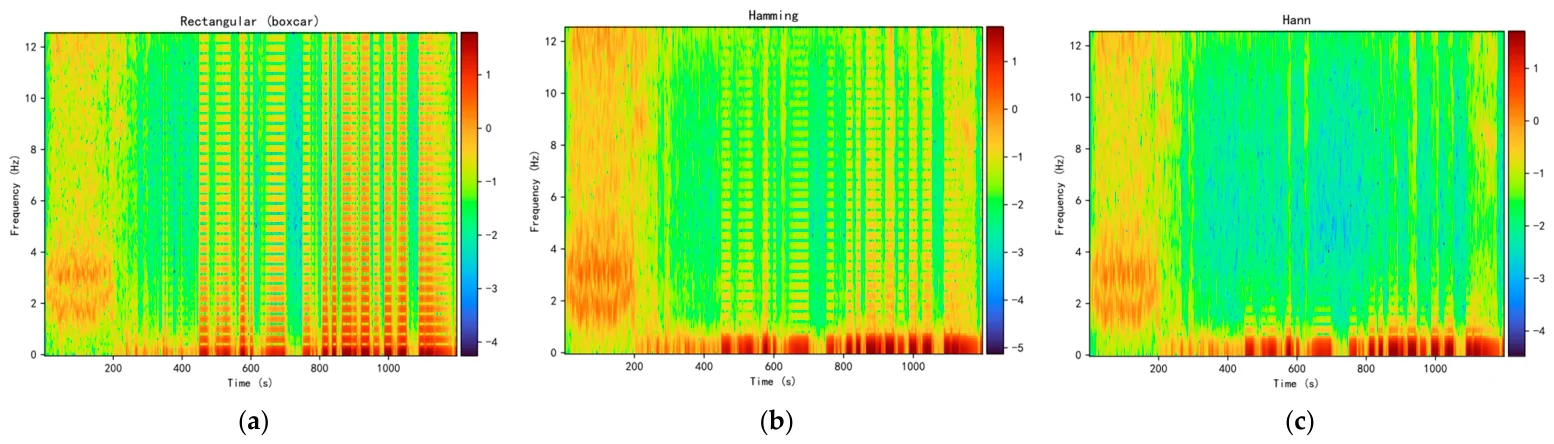
Comparison of flight parameter spectrograms obtained with different window functions. (**a**) Rectangular; (**b**) Hamming; (**c**) Hann.

To further verify the influence of window selection on feature extraction, measured spectrograms of the same flight parameter group under the three window functions are compared. As shown in Figure 4, the rectangular window produces strong spectral leakage due to endpoint amplitude jumps, generating obvious vertical stripe artifacts across the full frequency band, raising the background noise floor in non-maneuver segments, and weakening action boundaries. The Hamming window reduces part of the background noise, but energy diffusion remains at maneuver transients. By contrast, the Hann-window spectrogram shows a clearer maneuver background energy gradient, more complete suppression of broadband random interference in the high frequency region, and higher discriminability of low frequency maneuver signals in the time frequency domain.

To quantitatively evaluate the discriminative gain of different window functions for flight maneuver signals, a Low-to-High Frequency Contrast (LHC) metric $C_{lhc}$ is introduced as the evaluation criterion. The primary energy of flight maneuvers is concentrated in $\Omega_{l}\in \left[0,2\right]$ Hz, while environmental interference is predominantly distributed in $\Omega_{h}\in \left[4,f_{s}/2\right]$ Hz. The LHC is defined as the ratio of energy density in the target frequency band to that in the noise frequency band across the spectrogram:

(5) $$C_{lhc}=\frac{1}{K\left|\Omega_{l}\right|}\sum_{k}\sum_{\omega \in \Omega_{l}}10\log_{10}S_{i}(k,\omega )-\frac{1}{K\left|\Omega_{h}\right|}\sum_{k}\sum_{\omega \in \Omega_{h}}10\log_{10}S_{c}(k,\omega )$$

where K denotes the total number of spectrogram frames, $\left|\Omega_{l}\right|$ and $\left|\Omega_{h}\right|$ represent the number of frequency bins contained in the low- and high-frequency bands, respectively; and $S_{c}\left(k,\omega \right)$ is the power spectral density (i.e., the spectrogram value, defined in Equation (4)) of channel i at frame k and frequency ω. A higher $C_{lhc}$ value indicates a more pronounced average superiority of the low frequency maneuver energy over the high frequency noise in the logarithmic domain, suggesting a stronger discriminative capability of the window function for maneuver/noise classification.

To verify the generalizability of different window functions across multiple source parameters, five physical channels ($p,q,r,n_{y},n_{z}$) are processed with each of the three window functions using a fixed window length and DFT size. The resulting five-channel mean LHC values and frequency domain performance parameters are summarized in Table 2.

Table 2 shows that the Hann window is most suitable overall for constructing flight maneuver spectrograms. Although its main lobe width (0.293 Hz) is slightly wider and causes a small theoretical resolution loss, its side lobe suppression above 4 Hz reaches −97.2 dB, lower than those of the Hamming (−57.3 dB) and rectangular (−47.5 dB) windows. Under the fixed diagnostic STFT setting (*L* = 64, *H* = 32, *N* = 256), this side lobe attenuation reduces leakage of low frequency maneuver energy into high frequency regions, yielding five-channel mean LHC values of 14.86 dB (Hann), 13.54 dB (Hamming), and 12.80 dB (rectangular). Relative to the rectangular and Hamming windows, the Hann window increases mean LHC by 16.1% and 9.8%, respectively. Because the duration of flight maneuvers is usually much longer than the window length, the noise suppression benefit of the Hann window is more favorable for weak maneuver feature extraction. The Hann window is therefore selected as the basis for spectrogram construction, with the following time domain expression:

(6) $$w\left[n\right]=0.5\left(1-\cos\left(\frac{2\pi n}{L-1}\right)\right),0\leq n\leq L-1$$

The implementation of the STFT requires the optimization of three key parameters that govern the time frequency resolution, namely the window length, the hop size, and the DFT size, as discussed below.

(1) Window length  L

**Figure 5:**
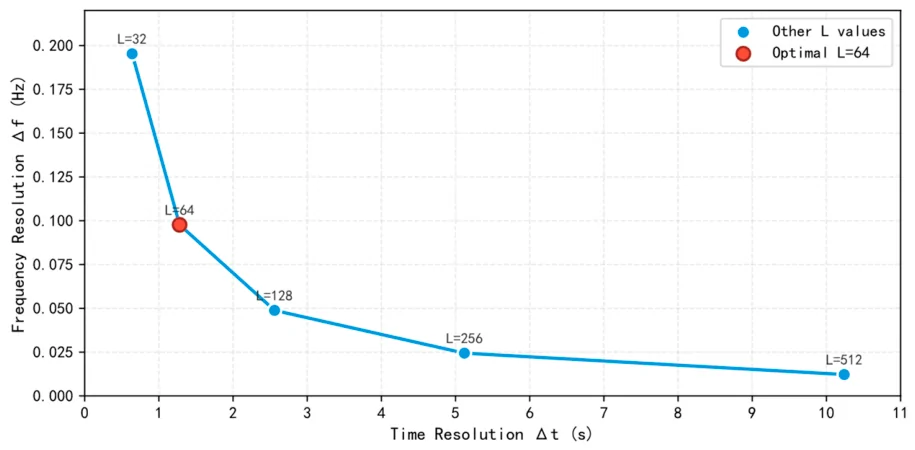
Time frequency resolution trade-off for window length *L* (Hann window, hop size = *L*/2).

The time frequency resolution of the STFT is jointly determined by the window length L, hop size H, and DFT size N, with the following relationships:

(7) $$\Delta f=\frac{f_{s}}{N},\Delta t=\frac{H}{f_{s}}$$

where the sampling rate $f_{s}$ is specified by the data. Increasing the window length improves frequency resolution but reduces temporal localization accuracy. A smaller hop size enhances information continuity between adjacent frames but increases computation. When the number of DFT points exceeds the window length, it only interpolates and refines the discrete frequency grid, without introducing new physical information. To determine the optimal configuration, window lengths L in {32, 64, 128, 256, 512} are scanned under a fixed Hann window and 50% overlap, and the joint trade-off between temporal resolution and frequency resolution is used as the selection criterion. The results are shown in Figure 5.

As shown in the Figure 5, frequency resolution and temporal resolution exhibit a typical trade-off as window length increases. A clear elbow appears at *L* = 64. Before this point, frequency resolution improves rapidly with increasing window length, while the cost in temporal resolution remains limited. Beyond *L* = 64, the marginal improvement in frequency resolution slows, whereas temporal resolution reaches more than 2.56 s, exceeding the reasonable lower bound of typical short duration maneuvers and increasing boundary ambiguity during maneuver entry and recovery. Therefore, *L* = 64 is selected as the best compromise.

(2) Hop size *H*

**Figure 6:**
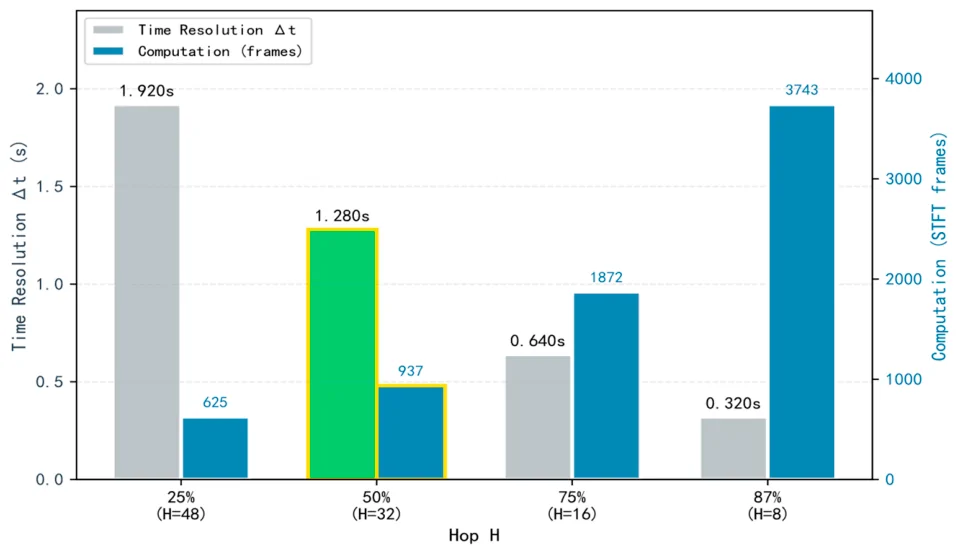
Comparison of computational load and time resolution under different overlap ratios. The green bar and yellow border highlight the selected 50% overlap configuration.

The temporal resolution and computational cost of STFT are directly constrained by the hop size *H*, or overlap ratio. A higher overlap ratio strengthens information continuity between adjacent short time windows and helps characterize the energy evolution at maneuver start and end more precisely. Excessive overlap, however, substantially increases temporal sampling redundancy and causes nonlinear growth in computational cost. Figure 6 shows the performance evaluation under different overlap ratios.

As the overlap ratio increases from 25% to 87%, the number of STFT frames rises sharply from 625 to 3743, imposing a heavier computational burden. Considering both computational load and temporal sampling requirements, 50% overlap is selected as the hop size configuration. This setting provides sufficient temporal sampling density for continuous maneuver boundaries while avoiding computational redundancy from overly dense frame sequences.

(3) Number of DFT points *N*

**Figure 7:**
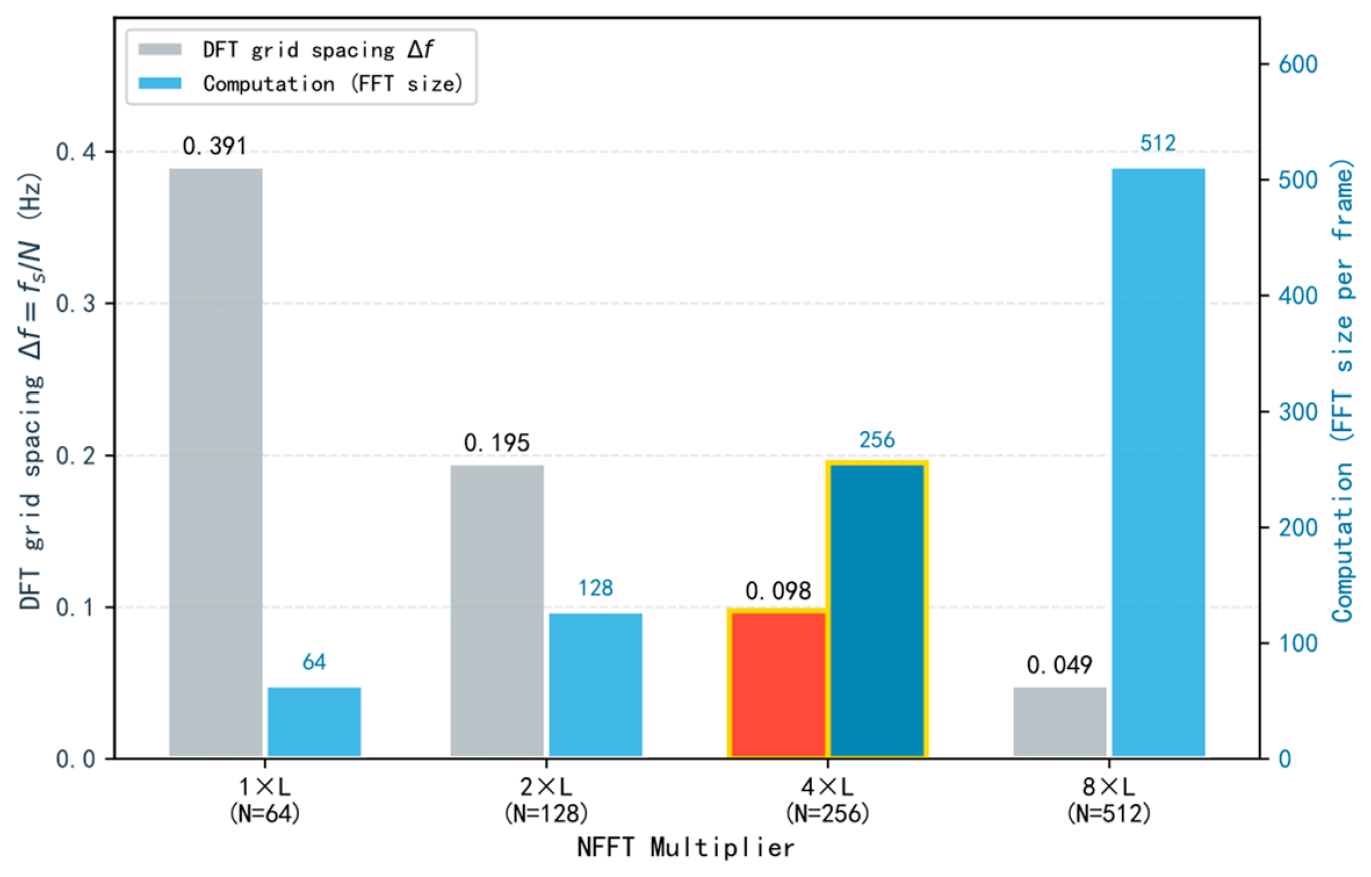
DFT grid spacing $\Delta f=f_{s}/N$ and computational load versus zero padding factor N/L (Hann window, L=64, H=32). The red bar and yellow border highlight the selected 4 × *L* (*N* = 256) configuration.

Zero padding refines the discrete frequency grid and improves observation precision for maneuver energy peaks. Figure 7 shows the influence of the zero padding factor (*N*/*L*) on spectral refinement and computational load. As the number of DFT points increases from 1*L* to 4*L* (*N* = 256), the interpolated representation of low frequency structures improves markedly. Further increasing to 8*L* makes the frequency grid denser, but the additional information mainly comes from interpolation and the computational cost rises sharply. Balancing finer DFT grid spacing against computational cost, we set the DFT size to four times the window length (N=256).

In summary, the STFT parameter configuration adopted in this paper is window length L=64 (Hann window), hop size H=32, and DFT size N=256. Under this configuration, the five-channel mean low-to-high frequency contrast for the Hann window ${\bar{C}}_{lhc}$ reaches 14.86 dB, effectively suppressing high frequency side lobe leakage.

## 4. Channel Quality Evaluation and Multi-Channel Spectrogram Fusion Enhancement

### 4.1. Maneuver Activation Ratio and Linear Contrast Ratio

The accuracy of flight maneuver recognition depends strongly on how sensitively each sensor channel represents maneuver excitation. Because the energy distributions of different maneuvers vary markedly across axes, quantifying each channel’s contribution is a prerequisite for efficient fusion. Let the total energy of the *i*-th channel at frame k be

(8) $$E_{i}\left[k\right]=\sum_{\omega }S_{i}\left(k,\omega \right)$$

To evaluate the temporal activity differences of each channel, the 70th and 30th percentiles of the energy sequence $\left\{E_{i}\left[k\right]\right\}$ are computed to define the maneuver-active frame set $\Lambda_{a}=\{k|E_{i}\left[k\right]>Q_{70}\}$ and the stationary frame set $\Lambda_{s}=\{k|E_{i}\left[k\right]<Q_{30}\}$, respectively. The Maneuver Activation Ratio (MAR) of channel *i*, denoted $M_{i}$, is defined as

(9) $$M_{i}=\frac{\frac{1}{\left|\Lambda_{a}\right|}\sum_{k\in \Lambda_{a}}E_{i}[k]}{\frac{1}{\left|\Lambda_{s}\right|}\sum_{k\in \Lambda_{s}}E_{i}[k]}$$

A higher $M_{i}$ indicates stronger energy separability between maneuver and stationary segments in that channel. Because maneuver feature energy is mainly concentrated in low frequency bands, whereas high frequency bands are often affected by structural vibration or sensor random noise, time domain energy proportion alone cannot fully evaluate time frequency feature quality. Therefore, a linear contrast ratio (LCR) is introduced. Unlike the LHC used for evaluating window function performance, LCR focuses on maneuver occurrence and quantifies the information contribution of each sensor channel within maneuver-active intervals, enabling dynamic data-driven weighting. $C_{\mathrm{lcr}}$ is defined as

(10) $$C_{\mathrm{lcr}}=\frac{\frac{1}{\left|\Omega_{l}\right|}\sum_{k\in \Lambda_{a}}\sum_{\omega \in \Omega_{l}}S_{i}(k,\omega )}{\frac{1}{\left|\Omega_{h}\right|}\sum_{k\in \Lambda_{a}}\sum_{\omega \in \Omega_{h}}S_{i}(k,\omega )}$$

This metric characterizes the spectral purity of a channel under maneuver activation. A higher $C_{\mathrm{lcr}}$ indicates that signal energy is more concentrated in the effective low frequency maneuver band and that the feature is cleaner; such a channel should therefore receive higher reliability during fusion.

**Figure 8:**
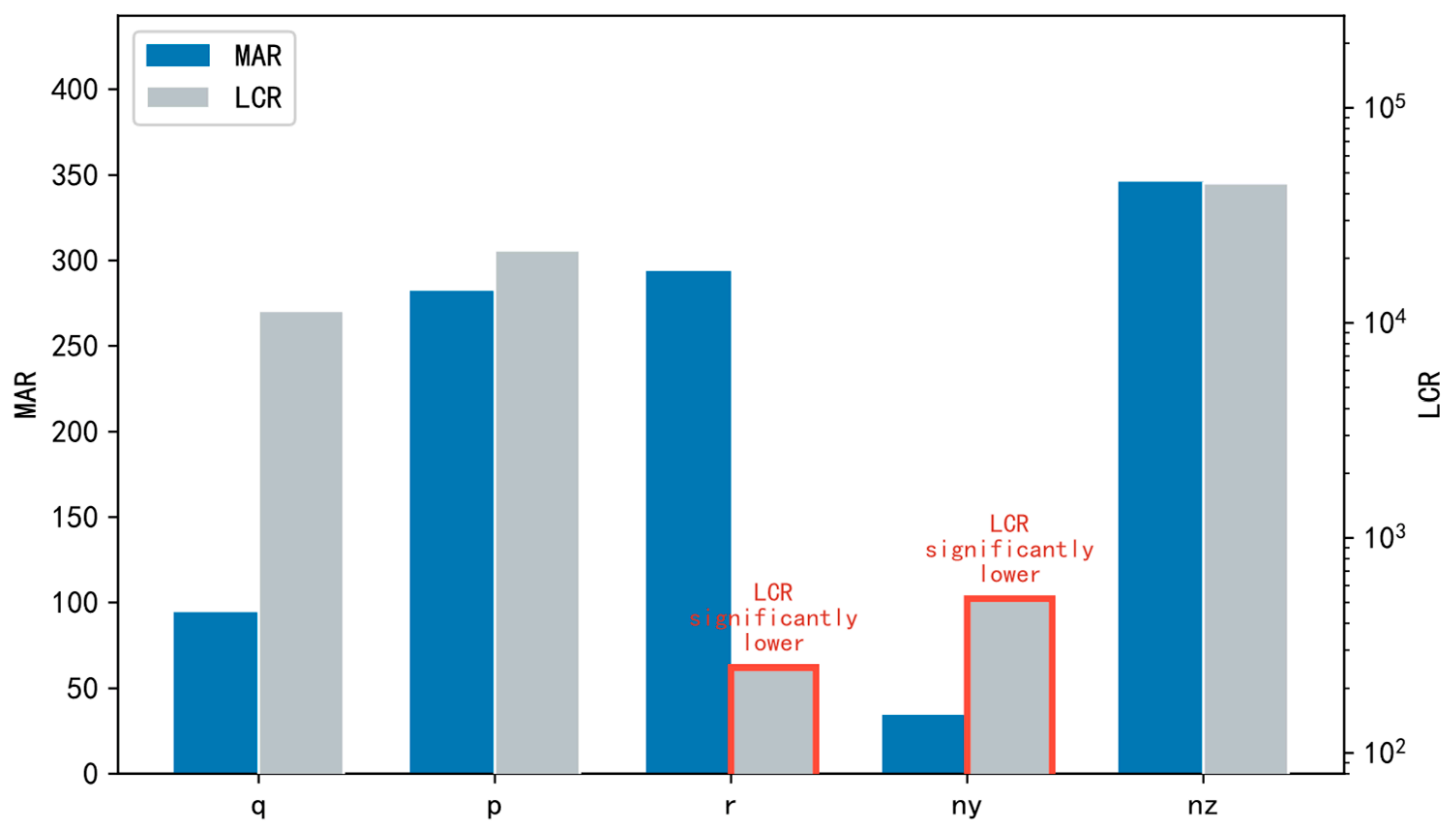
Comparison of Maneuver Activation Ratio (MAR) and Linear Contrast Ratio (LCR) across different channels.

Figure 8 presents the MAR and LCR comparisons for the five channels ($p,q,r,n_{z},n_{y}$). Both metrics are at relatively high levels for $n_{z}$ and p, marking them as the primary credible channels in the fusion process. In contrast, the r channel has a relatively high MAR but a significantly lower LCR, indicating that, while this channel exhibits energy enhancement during maneuvers, it is contaminated by substantial high frequency vibration components with insufficient spectral purity. This demonstrates that a single temporal activity metric (MAR) is inadequate to accurately characterize channel quality. Additionally, the $n_{y}$ channel ranks lowest on both metrics, indicating weak responsiveness to maneuver events. These comparisons validate the necessity of jointly employing MAR and LCR for channel quality evaluation, providing an objective basis for data-driven fusion weighting.

### 4.2. Spectral Entropy-Constrained Channel Weight Allocation

To allocate channel contributions reasonably in multi-channel spectrogram fusion, spectral entropy (SE) is introduced to characterize uncertainty in each channel’s time frequency energy distribution. An objective evaluation framework independent of empirical thresholds is constructed by combining MAR, LCR, and normalized spectral entropy to adaptively quantify feature reliability for each channel.

To characterize the information uncertainty of each sensor channel within the maneuver frequency band $\Omega \in \left[0,2.0\right]\mathrm{Hz}$, the spectrogram power $S_{i}\left(k,\omega \right)$ of channel i in the maneuver primary frequency band is first extracted and converted into a normalized frequency probability distribution $p_{i}\left(k,\omega \right)$ at frame k:

(11) $$p_{i}\left(k,\omega \right)=\frac{S_{i}\left(k,\omega \right)}{\sum_{\omega \in \Omega }S_{i}\left(k,\omega \right)}$$

Within the maneuver-active frame set $\Lambda_{a}$, the normalized entropy $SE_{i}$ is computed frame by frame and averaged to obtain the mean spectral entropy of channel i:

(12) $$SE_{i}=-\frac{1}{\left|\Lambda_{a}\right|}\underset{k\in \Lambda_{a}}{\sum }\left(\frac{1}{\log_{2}\left|\Omega \right|}\underset{\omega \in \Omega }{\sum }p_{i}\left(k,\omega \right)\log_{2}p_{i}\left(k,\omega \right)\right)$$

Due to the finite number of frequency bins $\left|\Omega \right|$, the normalization ensures that $SE_{i}\in \left[0,1\right]$. A smaller $SE_{i}$ value indicates that the channel energy is highly concentrated in the frequency domain, corresponding to a genuine maneuver response with a clear physical structure. Conversely, as $SE_{i}$ approaches 1, the energy distribution becomes more uniform and dispersed, suggesting the presence of high frequency structural vibrations or random noise. Consequently, $\left(1-SE_{i}\right)$ is defined as the energy concentration coefficient of the channel i.

Integrating the maneuver activation ratio $M_{i}$ (temporal activity), the linear contrast ratio $C_{\mathrm{lcr}}$ (frequency discriminability), and the energy concentration coefficient $\left(1-SE_{i}\right)$, the unconstrained initial fusion weight $w_{i}$ of channel i is constructed as

(13) $$w_{i}=M_{i}\cdot \log_{10}\left(C_{\mathrm{lcr}}+1\right)\cdot \left(1-SE_{i}\right)$$

where $\log_{10}\left(C_{\mathrm{lcr}}+1\right)$ is applied to compress the dynamic range of the contrast index. This fusion formula employs multiplicative modulation across three independent objective physical features. For channels contaminated by significant high frequency random noise, such as lateral and longitudinal acceleration, the $SE_{i}$ value is significantly higher, causing the $\left(1-SE_{i}\right)$ term to act as an attenuation factor that effectively suppresses the channel weight. Conversely, for core channels dominated by primary dynamic responses, such as the normal acceleration $n_{z}$, the highly concentrated low frequency energy drives all three metrics to high values, thereby achieving nonlinear weight enhancement. Finally, global linear normalization is performed on these data-driven initial scores to derive the final normalized channel weights ${\hat{w}}_{i}$ for spectrogram fusion:

(14) $${\hat{w}}_{i}=\frac{w_{i}}{\sum_{j=1}^{C}w_{j}}$$

**Figure 9:**
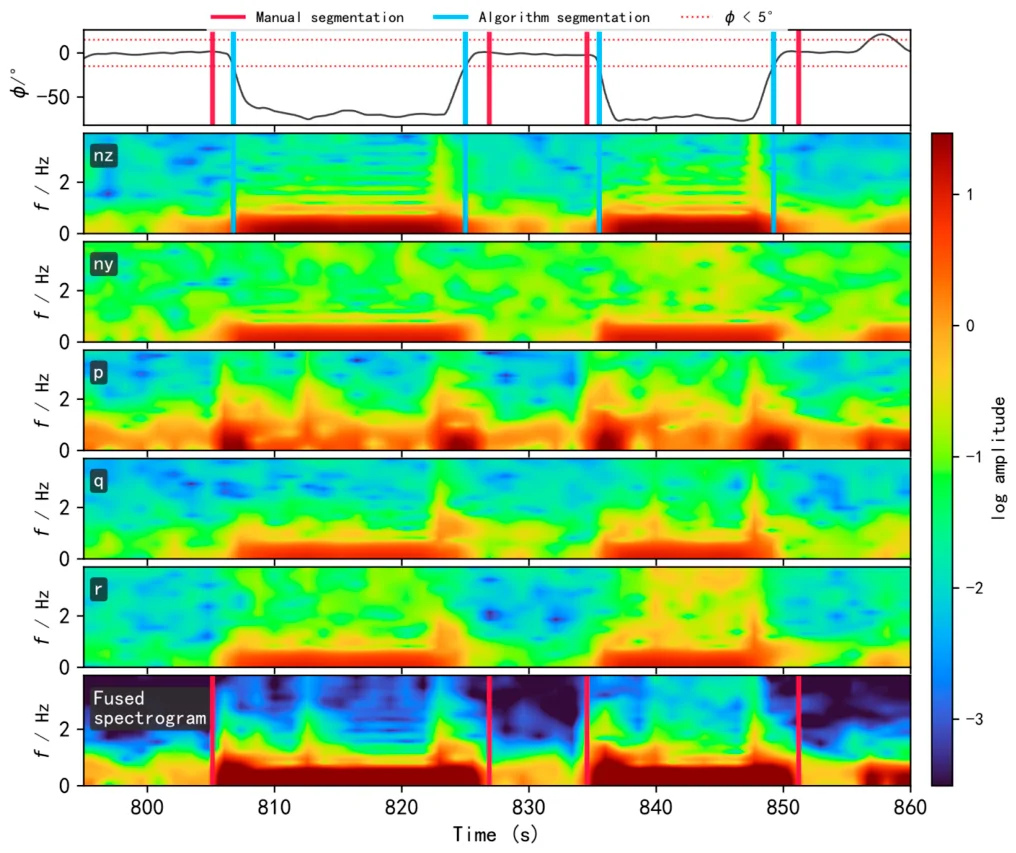
Comparison of single-channel spectrograms and the fused spectrogram with maneuver boundary annotations.

The data-driven weight allocation mechanism constrained by spectral entropy can adaptively attenuate the channel contributions of aerodynamic turbulence and high frequency structural vibrations based on the time frequency purity of each dimensional time series, thereby achieving high fidelity extraction and fusion of the core low frequency dynamic features of the airframe. Figure 9 presents a localized comparison between single-channel spectrograms and the fused spectrogram, with roll angle ϕ as the reference curve above; the blue vertical lines denote the maneuver boundaries determined by the single-channel $n_{z}$ spectrogram, whereas the red vertical lines represent the segmentation results obtained from the fused spectrogram. The figure shows that each individual channel exhibits low frequency energy enhancement during the maneuver interval but with pronounced heterogeneity in response characteristics: the $n_{z}$ channel can reflect load changes, but its boundaries are prone to fragmentation due to local energy valleys at action transitions; the p and q channels are sensitive to transient features but also introduce more high frequency interference; the r channel demonstrates relatively good continuity but with more diffuse background texture, making precise boundary localization difficult.

The spectral entropy-constrained weighted fusion mechanism quantifies time frequency purity across channels and realizes complementary integration of multidimensional information. It effectively integrates low frequency dynamic structures jointly supported by multiple channels while suppressing isolated disturbances in individual channels. Comparing the red and blue boundary lines shows that the fused spectrogram substantially improves robustness during maneuver recovery, avoids boundary shifts caused by single-channel energy decay, and aligns maneuver boundaries with the true maneuver response process. This high signal-to-noise time frequency structure provides an ideal input for subsequent soft gating and noise floor suppression, ensuring effective separation between maneuver regions and background noise.

### 4.3. Soft-Gating Enhancement, Background Noise Subtraction, and Fused Spectrogram Construction

Flight data commonly contain two types of interference: low amplitude broadband background noise generated during ground taxiing and engine warm-up before takeoff, and steady-state background noise in each channel during level flight. If full-duration spectrograms are directly fused with channel weights, these interferences are introduced equally into the fusion result and reduce the energy contrast between maneuver and non-maneuver segments. Therefore, soft gating and logarithmic noise floor subtraction are introduced before weighted fusion. We drive the gate with $n_{z}$ frame energy because normal overload best matches the load factor-rich maneuvers considered here, while noting that low load cases may be attenuated. Although $n_{z}$ also enters the weighted fusion, the soft gate is used only as a temporal reliability mask, not as an additional energy contribution.

(1) Normal overload soft gating

The normal overload channel is the most sensitive indicator of flight maneuvers, and its frame-level full-band power energy can effectively characterize the aircraft’s current motion state. The frame energy of the normal overload channel at frame k is defined as $E_{n_{z}}\left[k\right]=\sum_{\omega }S_{nz}\left(k,\omega \right)$. Using the 30th percentile ($P_{30}$) and 70th percentile ($P_{70}$) of the $E_{n_{z}}\left[k\right]$ sequence as the upper bound for the static state and the lower bound for the maneuver state, respectively, a piecewise linear soft gating function is constructed as follows:

(15) $$g[k]=\mathrm{clip}\left(\frac{E_{n_{z}}[k]-P_{30}}{P_{70}-P_{30}},0,1\right)$$

When $E_{n_{z}}\left[k\right]\leq P_{30}$, $g\left[k\right]=0$, corresponding to ground preparation or steady level flight phases, during which the spectrogram contribution is completely suppressed; when $E_{n_{z}}\left[k\right]\geq P_{70}$, $g\left[k\right]=1$, corresponding to intensive maneuver phases, where the spectrogram contribution is fully retained; within the transition interval, $g\left[k\right]$ increases linearly with energy, achieving a smooth transition between maneuver and non-maneuver states and avoiding temporal discontinuities introduced by hard threshold switching. Furthermore, to suppress gate jitter caused by abrupt inter-frame energy changes, a uniform moving average smoothing with a window of 5 frames is applied to $g\left[k\right]$ to obtain $\tilde{g}\left[k\right]$.

(16) $$\tilde{g}\left[k\right]=\frac{1}{5}\overset{2}{\underset{i=-2}{\sum }}g\left[k+i\right]$$

(2) Frequency-wise noise floor subtraction

To eliminate the influence of inherent noise floors across channels, logarithmic background noise estimation and subtraction are performed on a frequency-wise basis for each channel. For channel i at frequency ω, the noise floor estimate $N_{i}\left(\omega \right)$ is calculated by taking the 20th percentile of its logarithmic power sequence across all time frames:

(17) $$N_{i}(\omega )=\underset{k}{P_{20}}\left[\log_{10}S_{i}(k,\omega )\right]$$

where $P_{20}\left[\cdot \right]$ denotes the 20th percentile operator calculated over the frame index k. By subtracting the estimated noise floor from the logarithmic power spectrum and applying a non-negative constraint, the denoised power spectrum is obtained as follows:

(18) $${\hat{S}}_{i}(k,\omega )=\max(\log_{10}S_{i}(k,\omega )-N_{i}(\omega ),0)$$

(3) Fused spectrogram

Combining soft gating and noise floor subtraction, the final fused spectrogram $F\left(k,\omega \right)$ is defined as

(19) $$F\left(k,\omega \right)=\tilde{g}\left[k\right]\cdot \overset{C}{\underset{i=1}{\sum }}{\hat{w}}_{i}\cdot {\hat{S}}_{i}\left(k,\omega \right)$$

where ${\hat{w}}_{i}$ are the normalized fusion weights, and the sum is over all participating channels.

**Figure 10:**
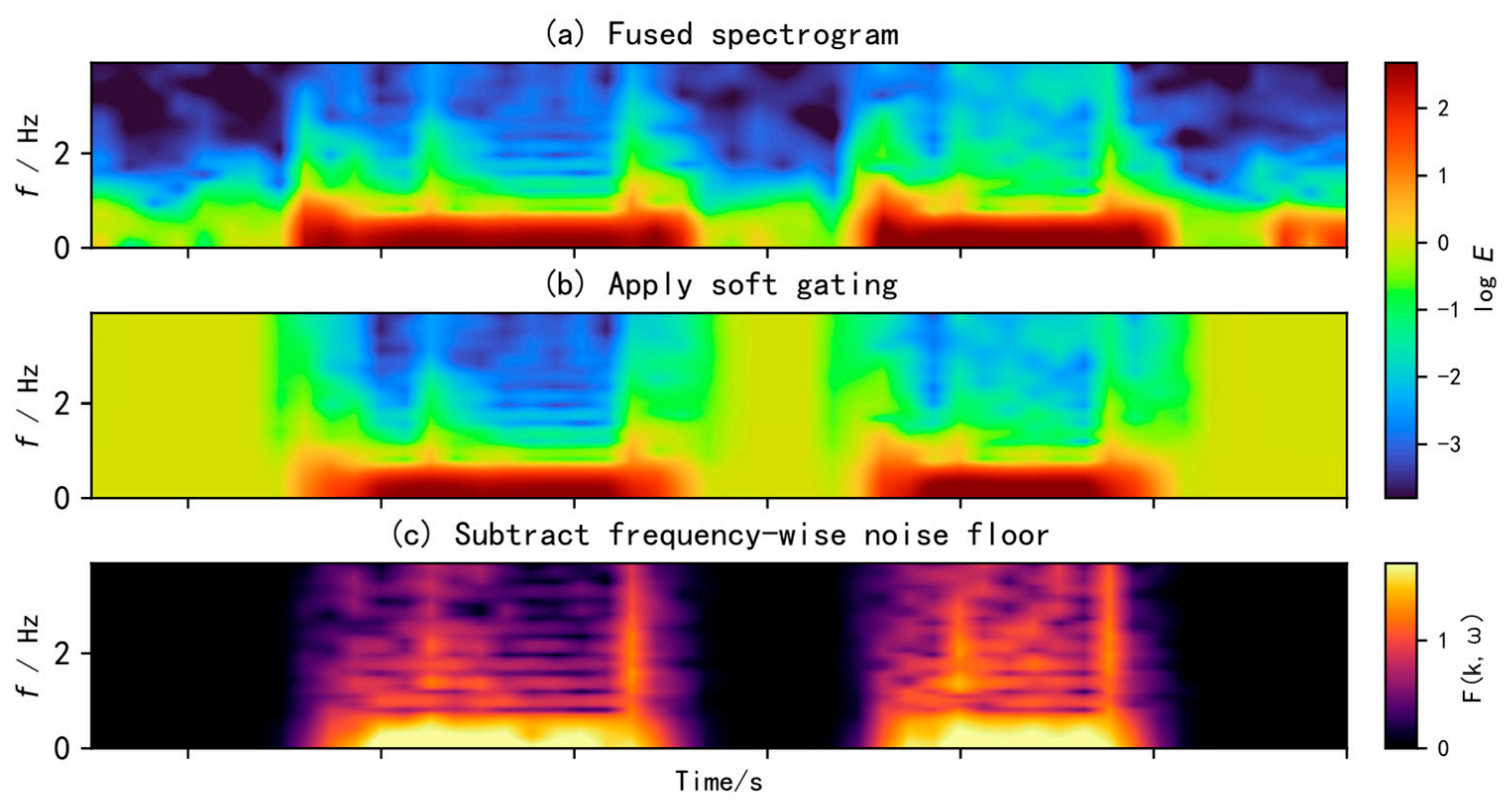
Comparison of the fused spectrogram before and after soft gating and noise floor subtraction.

Figure 10 compares fused spectrograms at different processing stages: Figure 10a shows the result after channel-weighted fusion only, whereas Figure 10b,c show the effects after introducing soft gating and frequency-wise noise floor subtraction. The comparison shows that relying only on channel weight allocation leaves obvious texture-like noise across all frequency bands in level flight background segments, indicating that a single weighting mechanism cannot fully suppress ground vibration and environmental interference. After soft gating, the spectrogram background energy in non-maneuver periods is effectively suppressed, and the start and end boundaries of maneuver intervals remain highly synchronized with the dynamic changes of the smoothed gating curve. Frequency-wise noise floor subtraction further suppresses residual frequency-dependent noise and highlights the low frequency, high energy structure under maneuvering conditions. These results show that the coordinated mechanism of soft gating and noise floor suppression significantly improves the time frequency separability between maneuver and background segments while preserving the integrity of maneuver responses.

## 5. Adaptive Maneuver Boundary Detection Algorithm

**Figure 11:**
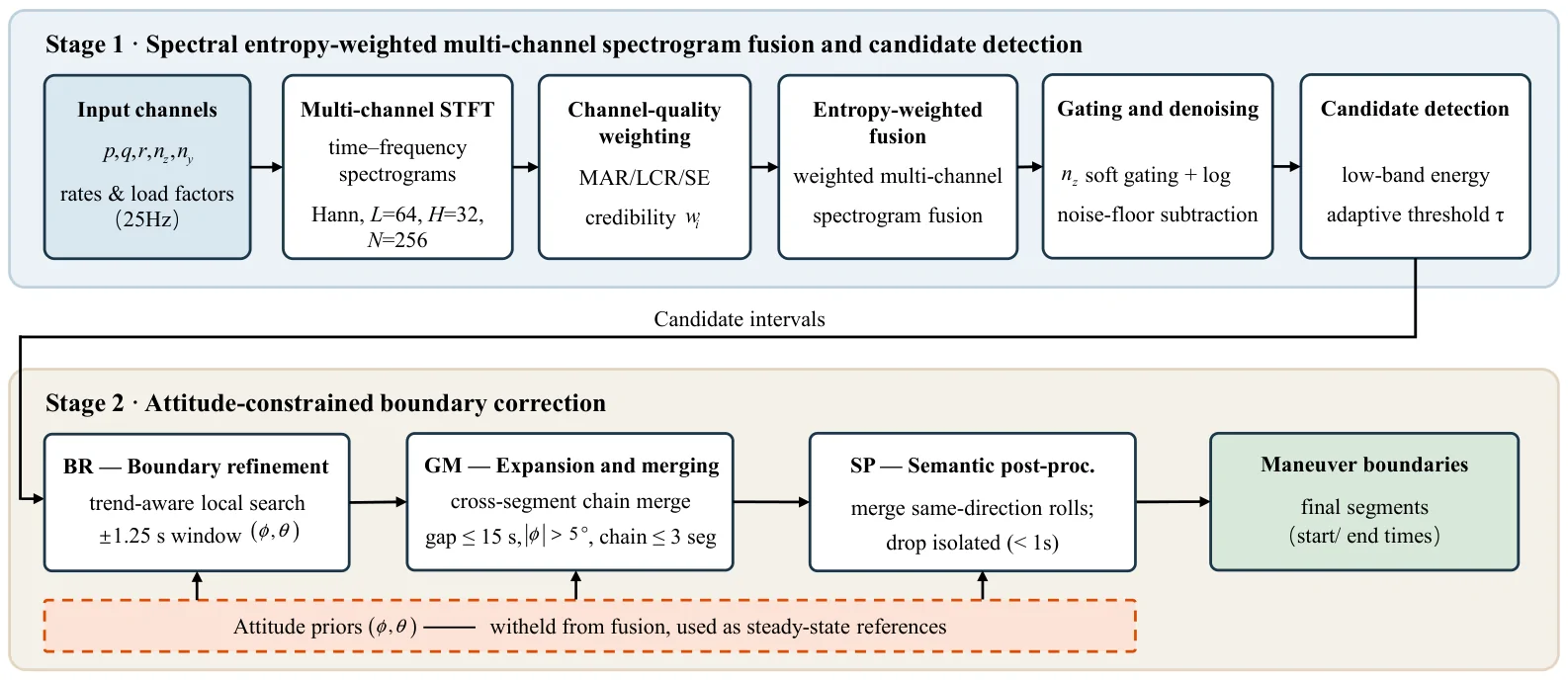
Overview of the proposed maneuver segmentation pipeline.

Having obtained an enhanced fused spectrogram, this section turns to adaptive extraction of maneuver intervals and subsequent boundary refinement. The procedure is organized into two stages: candidate detection from low frequency spectrogram energy, and attitude-constrained boundary correction that restores segment integrity using roll and pitch priors. Figure 11 provides a compact overview of this workflow.

In the candidate detection stage, five dynamic channels ($p,q,r,n_{z},n_{y})$ undergo STFT construction and entropy-weighted fusion (with soft gating and noise floor subtraction); candidate intervals are then obtained by adaptive thresholding of low frequency power. Attitude angles ϕ,θ are withheld from fusion and applied only in the correction stage (local refinement and cross-segment merging), yielding the final start/end boundaries.

### 5.1. Maneuver Indicator Sequence Construction

The primary dynamic features of flight maneuvers are concentrated in the low frequency region, where pitch, roll, and overload changes typically manifest as pronounced energy concentration in the 0–2 Hz band. Based on the fused spectrogram $F\left(k,\omega \right)$, the low frequency fused spectrogram is first extracted:

(20) $$S_{l}\left(k,\omega \right)=S\left(k,\omega \right),\omega \in \Omega_{f}$$

where $\Omega_{f}=\left(0,f_{h}\right]$, and the upper cutoff frequency is set to $f_{h}=2\;\mathrm{Hz}$.

Within the low frequency band, different frequency components contribute differently to maneuver state representation. Extremely low frequency regions usually correspond to sustained flight control and large-scale load changes, whereas relatively higher frequencies are more susceptible to structural vibration, random turbulence, and sensor noise. An inverse-frequency weighting factor $w_{f}\left(\omega \right)$ is therefore introduced to aggregate low frequency spectral energy for frame k:

Where the weighting factor is defined as $w_{f}\left(\omega \right)=1/\omega$. The DC bin (ω=0) is excluded from this sum; weights are computed only on positive frequency bins $\omega \in \left(0,f_{h}\right]$. The weight vector is then normalized to obtain ${\hat{w}}_{f}=w_{f}/\sum_{i}w_{f}\left(i\right)$.

(21) $$E_{w}\left[k\right]=\underset{\omega \in \Omega_{f}}{\sum }{\hat{w}}_{f}\left(\omega \right)\;S_{l}\left(k,\omega \right)$$

However, using only band-integrated energy to describe boundaries can easily diffuse transient edges during maneuver entry and recovery because of temporal smoothing induced by integration. To improve boundary localization accuracy, the maximum spectral peak within each frame is further introduced:

(22) $$E_{m}\left[k\right]=\underset{\omega \in \Omega_{f}}{\max}F_{l}\left(k,\omega \right)$$

Finally, to eliminate the interference caused by differences in physical dimensions and amplitude scales between distinct time frequency features, the two metrics are individually normalized using their respective maximum values and linearly fused to construct a unified maneuver indication sequence $E_{\mathrm{seg}}\left[k\right]$:

(23) $$E_{seg}\left[k\right]=\gamma \frac{E_{w}\left[k\right]}{\max\left(E_{w}\right)}+\left(1-\gamma \right)\frac{E_{m}\left[k\right]}{\max\left(E_{m}\right)}$$

where $\max\left(E_{w}\right)$ and $\max\left(E_{m}\right)$ denote the historical maximum values of the weighted power and the maximum spectral peak feature over the entire observation sequence, respectively; and γ represents the weighting coefficient for fusion. Unless otherwise stated, we use gamma = 1.0 for the soft gate exponent.

In this dual feature fusion architecture, the normalized integrated energy $E_{w}\left[k\right]$ maintains a steady response for sustained maneuvers such as long duration circling and smooth pull-up, preventing mid-segment breaks. The maximum spectral peak $E_{m}\left[k\right]$ substantially enhances edge sensitivity to short transient changes such as rapid rolls and instantaneous boundary entry. Their complementary fusion provides a feature basis for high precision and low false alarm boundary segmentation.

### 5.2. Adaptive Segmentation Threshold

**Figure 12:**
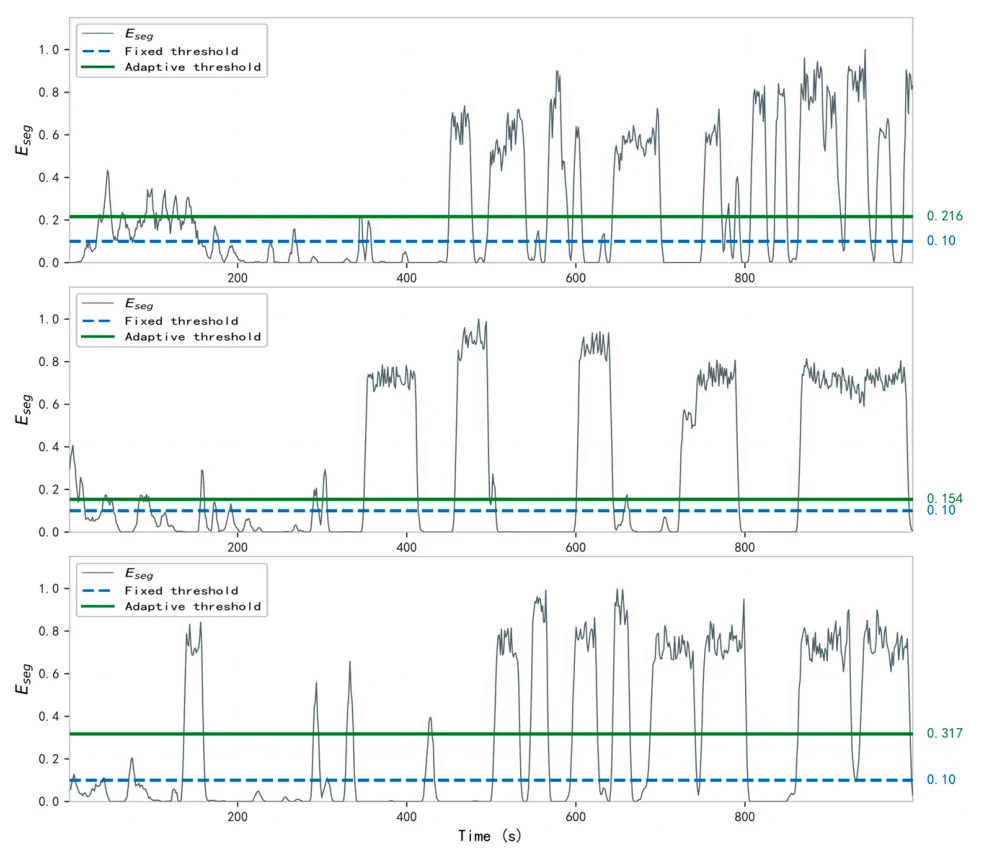
Comparison of segmentation results between fixed and adaptive thresholds across different flights.

Because flight mission complexity and airflow disturbance environments differ across sorties, a fixed global threshold can cause missed boundaries or over-segmentation. A local statistics-based adaptive threshold is therefore adopted. After the flight start frame $k_{0}$ (first frame with soft-gate > 0.20 for ≥5 consecutive frames) is determined, the local energy distribution of the maneuver indicator sequence $E_{seg}\left[k\right]$ is counted, and a dynamic threshold is defined by a percentile:

(24) $$T_{\mathrm{seg}}=\mathrm{Pct}\left(E_{seg}\left[k\right],\tau \right),k\geq k_{0}$$

where $\mathrm{Pct}\left(\cdot ,\tau \right)$ denotes the τ-th percentile operator, with τ=70% selected on the validation sorties only and then frozen for test and external evaluation. Compared to a fixed threshold, the adaptive percentile strategy dynamically adjusts the decision boundary based on the overall energy level of the flight data. Specifically, when the flight environment is relatively stable, the threshold automatically decreases to enhance sensitivity to weak maneuvers; conversely, in high noise environments, the threshold is elevated accordingly to mitigate false alarms. Figure 12 illustrates the segmentation performance of fixed versus adaptive thresholding strategies across different flight segments. It is evident that the background energy distribution fluctuates significantly across segments, rendering a single fixed threshold (0.10) inadequate for varying mission environments. In the first segment, the fixed threshold leads to extensive background noise being misclassified as maneuvers; similarly, in the third segment, high background noise levels increase the risk of false positives. In contrast, the adaptive threshold strategy dynamically adjusts the decision criteria (0.216, 0.154, 0.317) based on the distribution characteristics of $E_{seg}$ within each segment. This adaptive mechanism effectively filters out environment-specific interference while avoiding the under-segmentation or over-segmentation issues associated with fixed thresholds, thereby improving robustness to within-type background-energy variation across sorties of the same aircraft family.

### 5.3. Morphological Post-Processing and Boundary Optimization

Based on the dynamic threshold $T_{seg}$, the maneuver indication sequence is binarized into a mask $M\left[k\right]$, where $M\left[k\right]=1$ if $E_{seg}\left[k\right]>T_{seg}$, and 0 otherwise. Given that $M\left[k\right]$ often contains single-frame discontinuities or isolated noise points, morphological operators are employed to restore temporal continuity. To address these non-continuous defects and ensure that the segmented boundaries align with flight dynamics priors, a two-stage post-processing workflow is adopted. First, a one-dimensional morphological closing operation is applied to eliminate fractures, jitters, and noise, thereby restoring temporal continuity. Subsequently, trend-aware fine-tuning is performed based on attitude stability priors to refine the boundaries.

To repair single-frame holes, edge spurs, and isolated noise caused by discontinuous energy responses in the maneuver mask $M\left[k\right]$, one-dimensional morphological closing is applied to smooth maneuver regions. Specifically, dilation is first used to connect instantaneous breaks between maneuver fragments:

(25) $$M_{d}\left[k\right]=M\left[k\right]⊕B_{d}$$

Erosion is then used to remove local spurs and redundant noise introduced during dilation:

(26) $$M_{e}\left[k\right]=M_{d}\left[k\right]⊖B_{e}$$

In the formulas, $B_{d}=2$ and $B_{e}=1$ frames represent the defined structuring elements, the lengths of which determine the neighborhood scale for the morphological operations. The essence of the closing operation (dilation followed by erosion) lies in its ability to effectively fill transient fractures within maneuver sequences and smooth the jitter at boundary transitions by sliding the structuring elements across the signal, thereby reinforcing the temporal integrity and continuity of the maneuver segments.

Following the morphological processing, the connected component labeling algorithm is applied to extract candidate maneuver segments. Let $\left[t_{s}^{k},t_{e}^{k}\right]$ denote the start and end times of the *k*-th candidate segment. To filter out false maneuvers caused by noise, a minimum duration threshold $T_{min}=3\;s$ is established at the energy mask stage. Any segment satisfying $t_{e}^{k}-t_{s}^{k}<T_{min}$ is considered transient noise and discarded. Furthermore, considering the existence of extremely short transition pauses between maneuvers, a merging threshold $T_{gap}=3.8\;s$ is defined for short pauses in the mask stage. If the time interval between two adjacent segments is less than $T_{gap}$, they are automatically merged into a single continuous maneuver segment. Upon completion of these processes, the candidate maneuver segment set $A_{0}$ is obtained:

(27) $$A_{0}={\left\{\left[t_{s}^{k},t_{e}^{k}\right]\right\}}_{k=1}^{K},K\leq K_{0}$$

where $K_{0}$ denotes the total number of initially detected maneuver segments. Although this processing workflow ensures the continuity of time frequency energy boundaries, the smoothing effect inherent in the energy response may cause the detected boundaries to deviate from the true control critical points. Consequently, incorporating attitude information is essential to achieve further refinement and precise correction of these boundaries.

Temporal morphological refinement effectively removes instantaneous breaks and isolated noise in candidate intervals, but segmentation based on time frequency energy still contains two systematic biases. First, group delay caused by STFT windowing makes the energy response lag behind the actual control input, causing segmentation boundaries to shrink inward relative to the true critical points. Second, a pure energy criterion cannot identify instantaneous attitude zero crossings during sustained large amplitude roll actions such as circling, and may split a single continuous maneuver into multiple fragments. To remove these biases, roll angles $\phi \left(t\right)$ and pitch angles $\theta \left(t\right)$ are introduced as refinement parameters. Local trend refinement, cross-segment chain merging, and same-direction semantic merging are then used to correct maneuver boundaries precisely.

Given the dynamic characteristic that flight maneuvers typically “start and end in level flight,” a hard constraint for level flight steady state can be defined as $\left|\phi \left(t\right)\right|<\phi_{\mathrm{thr}}$, where $\phi_{\mathrm{thr}}=5{}^{\circ}$ serves as the roll threshold. To address the failure of this criterion at maneuver exits due to the aircraft maintaining a non-zero pitch steady state, a trend-aware scoring function $\delta \left(t\right)$ is constructed:

(28) $$\delta \left(t\right)=\left|\varphi \left(t\right)\right|+w_{\theta }\cdot P_{\theta }\left(t\right)$$

In this expression, $w_{\theta }$ represents the weight of the pitch penalty term, which is determined to be 0.3 via grid search on the validation set. This weighting serves to balance the contributions of roll and pitch attitude deviations, thereby optimizing the trade-off between suppressing false maneuvers and maintaining sensitivity to legitimate maneuver boundaries. $P_{\theta }\left(t\right)$ is derived from the statistical characteristics of θ within a local sliding window of ±0.5 s. Specifically, if the variance satisfies $\sigma_{\theta }^{2}<4$ and the mean absolute value $\left|\bar{\theta }\right|>3$, the aircraft is considered stabilized at a non-zero pitch attitude, and $P_{\theta }\left(t\right)$ is set to zero; otherwise, the instantaneous absolute value $\left|\theta \left(t\right)\right|$ is adopted as the penalty. The function $\delta \left(t\right)$ quantifies the overall deviation of the attitude from the level flight steady state, where a smaller value indicates a state closer to ideal level flight.

For long duration rolling maneuvers, if adjacent segments satisfy the criteria for consistent directionality and significant roll amplitude, they are associated through a semantic merging mechanism to eliminate over-segmentation caused by transient zero crossings within the maneuver. Furthermore, based on the physical properties of flight maneuvers, anomalous segments that are too short or fail to meet the significance requirements for roll amplitude are filtered out, and fragments separated by negligible temporal gaps are cleared. Finally, the optimized maneuver boundary set $A^{*}$ is output:

(29) $$A^{*}={\left\{\left[{\tilde{t}}_{s}^{k},{\tilde{t}}_{e}^{k}\right]\right\}}_{k=1}^{K^{*}}$$

where $K^{*}$ denotes the final number of maneuver segments. The boundaries of this set are locked to the local minima of the scoring function $\delta \left(t\right)$ within a physically feasible range, ensuring precise alignment with the true critical points where the aircraft transitions from steady-state level flight to maneuver. This effectively eliminates the systematic bias introduced by the group delay in time frequency energy responses. Meanwhile, leveraging the semantic merging mechanism for consistent maneuver directions, this method preserves the structural integrity of long duration maneuvers, such as sustained circling and turns, thereby overcoming the over-segmentation challenges inherent in relying solely on energy-based criteria.

**Figure 13:**
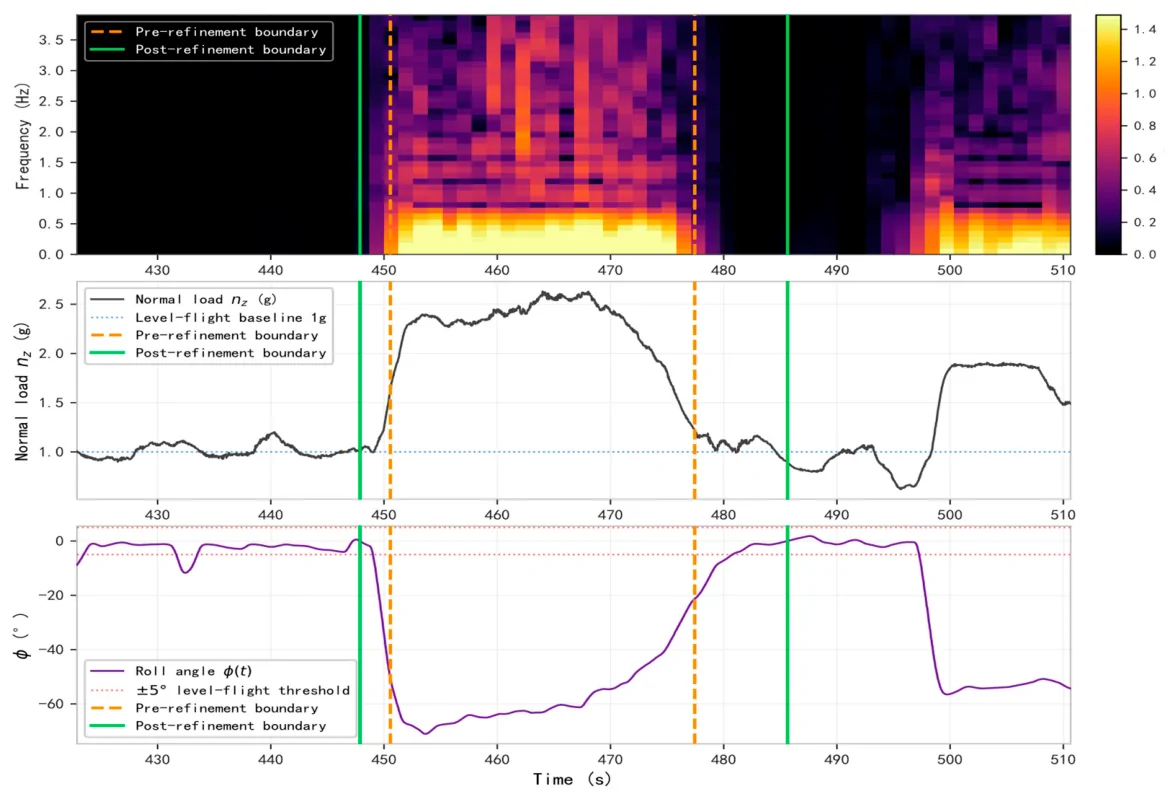
Comparison of maneuver boundary refinement based on trend-aware correction.

Figure 13 presents a comparison of boundary fine tuning results before and after processing, where orange dashed lines indicate candidate boundaries after morphological refinement, and green solid lines represent the final result after trend-aware fine tuning. The original candidate boundaries show a pronounced systematic inward bias due to the group delay of energy responses relative to control inputs. After trend-aware correction, the refined boundaries can sensitively capture the critical features of attitude deviation from the level flight steady state, substantially reducing the temporal lag introduced by time frequency analysis and improving maneuver boundary localization accuracy.

## 6. Experimental Validation and Comparative Analysis

### 6.1. Dataset and Evaluation Criteria

The primary experimental data were obtained from the flight data recording system of a single-engine fighter aircraft (sortie IDs SE_001–SE_020), comprising synchronous multi-parameter time series from 20 independent flight sorties recorded at 25 Hz. The dataset incorporates seven core parameters characterizing the spatial motion state of the airframe: pitch rate q, roll rate p, yaw rate r, normal load factor $n_{z}$, lateral load factor $n_{y}$, roll angle ϕ, and pitch angle θ. The cumulative effective flight duration is approximately 16.0 h, with individual sortie durations ranging from 37.0 to 63.9 min. Each sortie covers the complete mission profile from taxiing to landing and was conducted at a military airfield under standard visual meteorological conditions as part of routine advanced pilot training, in which pilots executed a predetermined sequence of maneuvers at altitudes between 1500 m and 5000 m with no restrictions on execution style, thereby preserving natural inter-pilot and inter-repetition variability in mission complexity, atmospheric turbulence, and pilot operational style. Ground truth maneuver boundaries were established via a double-blind protocol: initial boundaries were independently delineated by experienced analysts, followed by a secondary independent review; segments with boundary discrepancies exceeding 2 s or with ambiguous segmentation entered joint adjudication, yielding a ground truth set of $A_{T}={\left\{\left[t_{s,i},t_{e,i}\right]\right\}}_{i=1}^{K}$ 461 high confidence maneuver intervals. The single-engine data partition is structured as follows: training sorties SE_001–005 (5 sorties), validation sorties SE_006–017 (12 sorties), and held-out test sorties SE_018–020 (3 sorties). Primary supervised comparisons are reported on the held-out test sorties, with results across all 20 sorties additionally reported as reference for ablation analysis. Performance is evaluated using per-sortie means, standard deviations, and bootstrap 95% confidence intervals.

Seven maneuver categories are defined covering the core aerobatic and tactical repertoire of the training syllabus: Loop, Half Cuban Eight, Horizontal Figure Eight, High Speed Roll, Turn, Circling, and Combined Maneuver, where the last category refers to instances in which two or more basic maneuver types are executed in immediate succession without a level flight recovery interval. An independent twin-engine fighter corpus (sortie IDs TE_001–TE_010, 221 annotated intervals), collected using the same seven-channel parameter recording system at 25 Hz but under completely independent conditions in terms of aircraft type, pilots, airfield, and sortie planning, is reserved for external cross-platform generalization testing. The interval counts and class proportions for both the primary corpus and the external validation set are summarized in Table 3.

To comprehensively and objectively quantify the maneuver extraction performance of the algorithm in complex time series records, a multidimensional evaluation system is constructed based on three metrics: candidate detection capability, interval coverage integrity, and boundary localization accuracy. Let $A_{C}=\left\{I_{j}\right\}$ denote the set of algorithm-detected segments and $A_{T}=\left\{I_{i}\right\}$ be the set of ground truth segments. The evaluation utilizes a greedy one-to-one matching strategy based on the Intersection over Union (IoU). All potential matching pairs are ranked in descending order of IoU, and those satisfying the following condition are identified as True Positives (*TP*):

(30) $$\mathrm{IoU}\left(I_{j},\;I_{i}\right)=\frac{|I_{j}\cap I_{i}|}{|I_{j}\cup I_{i}|}\geq 0.5$$

Algorithm-detected segments that fail to achieve a match are classified as False Positives (*FP*), while unmatched ground truth segments are counted as False Negatives (*FN*). The candidate detection performance is quantified using Precision $P_{r}=TP/\left(TP+FP\right)$, Recall $R_{e}=TP/\left(TP+FN\right)$, F1-score $F_{1}=2P_{r}R_{e}/\left(P_{r}+R_{e}\right)$, and the total number of detected segments, which intuitively reflects the degree of over-segmentation.

Interval coverage integrity is assessed via two mean-based metrics. The first is the average IoU of all TP matching pairs, measuring the temporal alignment quality of correctly detected segments. The second is the Global Maximum Coverage score (${\bar{\mathrm{IoU}}}_{\max}$), defined as

(31) $${\bar{\mathrm{IoU}}}_{\max}=\frac{1}{\left|A_{T}\right|}\underset{i}{\sum }\underset{j}{\max}\mathrm{IoU}\left(I_{i},I_{j}\right)$$

This metric relaxes the strict one-to-one matching constraint, aiming to evaluate the theoretical maximum coverage capability of the algorithm across all ground truth intervals. Finally, boundary localization accuracy is quantified using the Mean Absolute Error of the start boundaries (${\mathrm{MAE}}_{s}$) and end boundaries (${\mathrm{MAE}}_{e}$), along with their mean ${\mathrm{MAE}}_{b}=\left({\mathrm{MAE}}_{s}+{\mathrm{MAE}}_{e}\right)/2$, which serves as a single index for assessing the overall localization bias. For the complete method on all 20 single-engine sorties, the matched IoU mean is 0.917, and the mean boundary ${\mathrm{MAE}}_{b}$ is 1.477 s (start 1.484 s, end 1.471 s). With hop H = 32 at 25 Hz (frame step 1.28 s), these ${\mathrm{MAE}}_{s}$ lie near one frame step; trend-aware refinement operates on the full-rate 25 Hz attitude signals (phi, theta), which can in principle localize boundaries finer than the STFT hop. Absolute errors should therefore be read against both the 1.28 s hop floor and the 2 s annotation disagreement tolerance.

### 6.2. Comparative Experiment on Maneuver Segmentation

To verify the effectiveness of the proposed maneuver segmentation method, representative comparison algorithms are designed for horizontal evaluation under a common IoU ≥ 0.5 protocol; their technical characteristics are listed in Table 4. Baselines A–G differ mainly in sensor channel usage and in time domain versus time frequency feature modeling, and none include attitude-constrained boundary correction (H = G + BR/GM/SP). The design targets the respective roles of multi-channel extension, spectrogram-based representation, and entropy-weighted fusion. E (FM-detector–style) and F (DTS-Net) are added as learning references: the former uses proposal-based end-to-end interval detection on multivariate series, and the latter uses deep temporal segmentation with interval extraction; both employ seven channels, are trained on training/validation sorties only, and are tested under the same matching rule as A–H.

**Figure 14:**
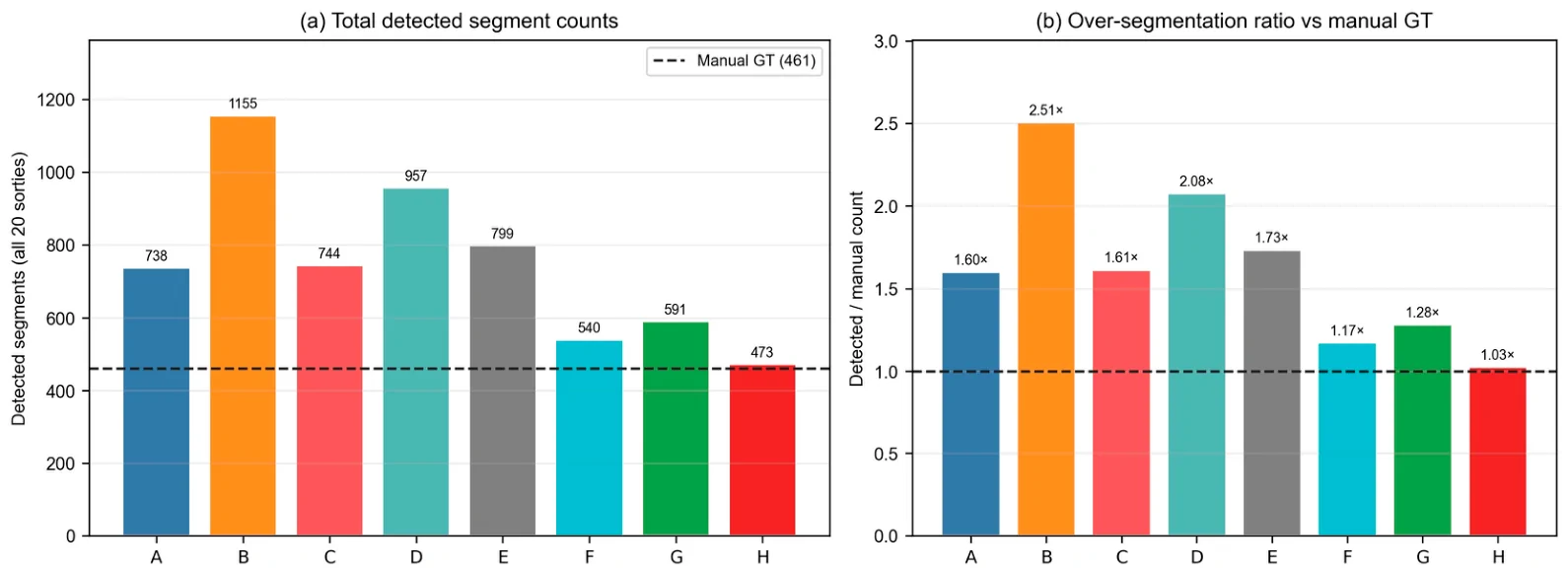
Comparison of detected maneuver counts for different segmentation algorithms across flights. (**a**) Total detected segments; (**b**) ratio to the manual ground truth count (461).

Figure 14 reports maneuver segment count statistics on all 20 single-engine sorties (SE_001–SE_020)—the full labeled corpus—not on the held-out test split alone. Figure 14a shows the total number of detected segments summed over these 20 flights; Figure 14b shows the ratio to the manual ground truth total (461 intervals). This view is intended as a complementary reference for the over-segmentation scale rather than as the primary supervised metric report. All methods exceed the annotated count to varying degrees. B is the most severe (1155 segments, 2.51× GT), indicating that multi-channel time domain energy without frequency selectivity is highly sensitive to broadband background activity. A and C yield similar totals (738 and 744, 1.60× and 1.61×) but reflect different mechanisms: amplitude thresholding in A tends to miss sustained low amplitude maneuvers, whereas local variance detection in C responds strongly to transients yet fragments smooth sustained events. D produces an even higher total (957, 2.08×), showing that single-channel STFT energy with dual low/high frequency gating does not by itself stabilize segment scale. Learning baselines E (FM-detector, 799, 1.73×) and F (DTS-Net, 540, 1.17×) reduce fragmentation relative to several DSP methods but still depart from the annotated scale. The fusion front end G lowers the total to 591 (1.28×), closest among the detection-stage comparators, consistent with MAR/LCR/entropy weighting and gating suppressing count inflation in most flights (panel (b)). The complete pipeline H aligns best with manual annotations (473, 1.03×), indicating that attitude-constrained correction further restores segment integrity beyond G alone.

**Figure 15:**
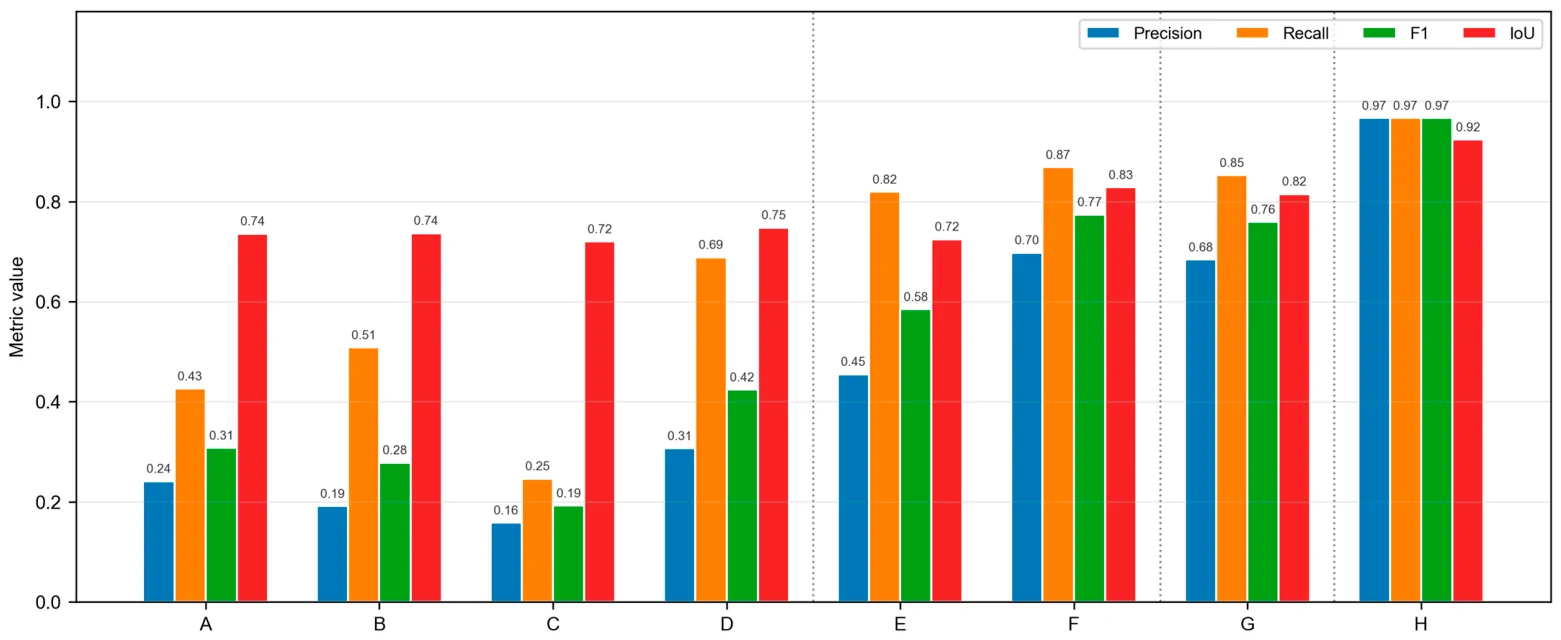
Performance comparison of different segmentation algorithms.

Figure 15 presents Precision, Recall, F1, and matched IoU on the held-out test sorties only (SE_018–SE_020, three sorties) under IoU ≥ 0.5 interval matching; training and validation sorties (SE_001–SE_017) are excluded. This figure provides the primary quantitative comparison used for headline supervised performance. The results do not improve monotonically with channel or model complexity, reflecting a trade-off between sensitivity and false alarm control. C remains weakest on the test split (F1 0.19); its matched IoU (0.72) is moderate, but substantial missed detection limits overall F1, showing that local overlap on matched pairs and global detection completeness are distinct dimensions. B slightly increases recall over A but sharply reduces precision because of over-segmentation. D improves recall (0.69) over A/C, while precision stays low (0.31), so single-channel spectrogram energy still admits stable flight false positives. F (DTS-Net, F1 0.77) and G (fusion front-end, F1 0.76) outperform E (FM-detector, F1 0.58) and all DSP baselines A–D. Relative to D, G improves precision by 0.38 and recall by 0.16, consistent with multi-channel quality weighting and background suppression rather than indiscriminate sensitivity gain. H reaches F1 0.967 (≈ 0.97) (Precision/Recall ≈ 0.97, IoU 0.92) on the same test split, confirming that BR/GM/SP correction yields the decisive margin after fusion-based detection; the remaining segment count excess of G on the all-20 view (Figure 14) is largely resolved at the test split metric level by H.

Across all 20 single-engine sorties, the proposed method yields a per-sortie F1 of 0.941 ± 0.056 (mean ± std; bootstrap 95% CI [0.915, 0.963]). Paired Wilcoxon signed-rank tests on the 20 sortie-level F1 scores show significant improvements over Algorithm G (formerly E), Algorithm D, DTS-Net, and the FM-detector (all $p=1.9\times 10^{-6}$; mean ΔF1 = 0.198/0.490/0.141/0.322, respectively).

### 6.3. Ablation Experiment

Staged BR/GM/SP ablations build the complete method H from the fusion front end G (Table 3). These experiments isolate mechanism contributions at the boundary correction stage and do not include the learning baselines E (FM-detector) and F (DTS-Net). On the full single-engine corpus, G already achieves strong candidate-level recall, but its total detected count (591) still exceeds manual annotations (461), and long continuous maneuvers remain fragmented when low frequency energy valleys split the mask. Three attitude-constrained modules are therefore added sequentially on top of G: local boundary refinement (BR), cross-segment expansion and chain merging (GM), and semantic post-processing (SP). The resulting progressive variants are G + BR, G + BR + GM, and G + BR + GM + SP (equivalent to H). BR searches, within a local window of ±1.25 s around each candidate endpoint, for a trend-aware stable point based on roll and pitch and adjusts boundaries in place within a single segment. GM allows cross-segment expansion and chain-merges adjacent candidates when the inter-segment gap is within 15 s, roll angle stays above 5° over the gap, and the merge chain length does not exceed three segments. SP applies semantic post-processing: merging same-direction continuous roll segments, filtering isolated segments longer than 12 s with peak |roll| < 5°, and removing fragments shorter than 1 s. The role of each stage is summarized in Table 5.

**Figure 16:**
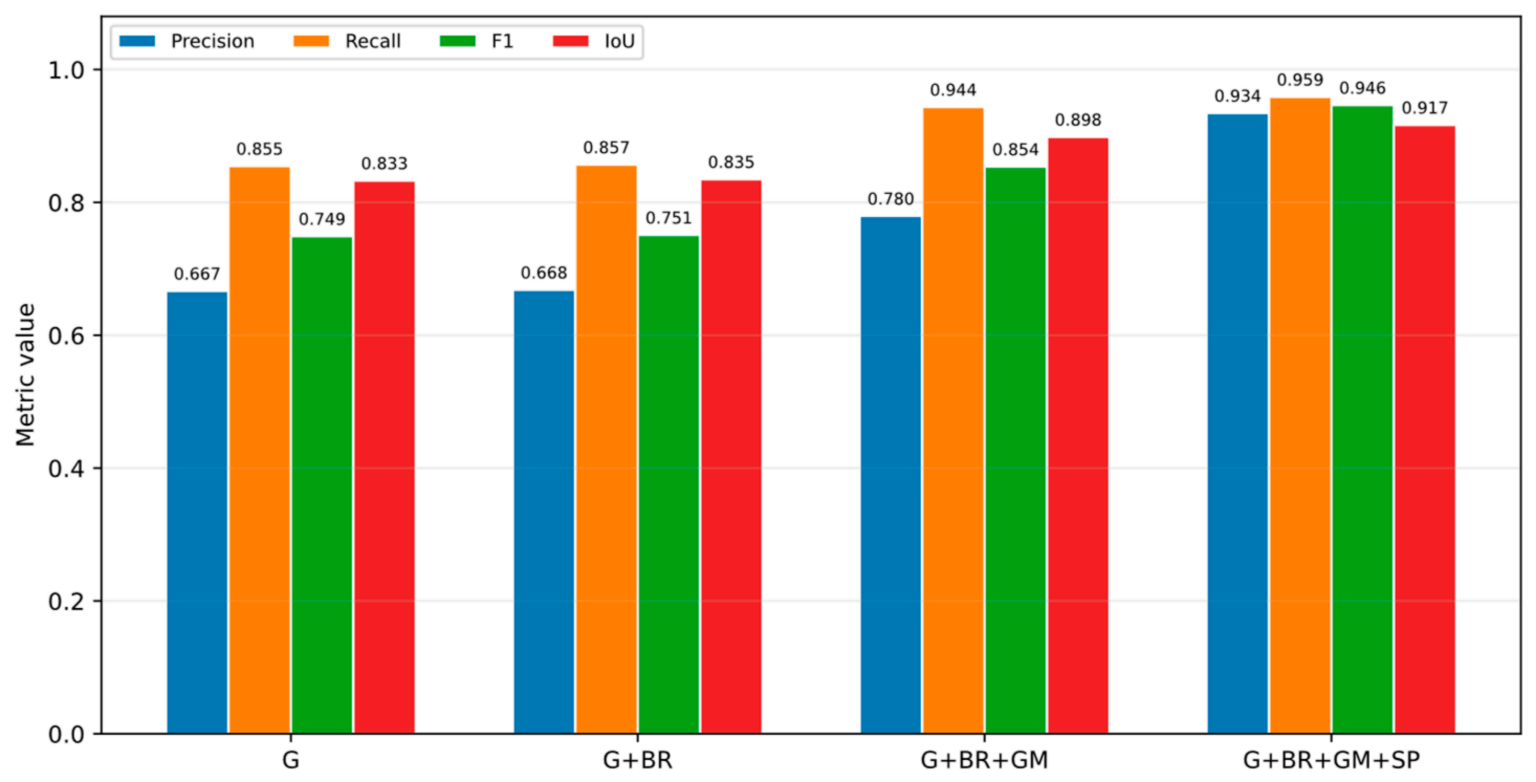
Performance comparison of different ablation variants.

Figure 16 shows the staged changes in Precision, Recall, F1-score, and IoU for the four ablation algorithms. After adding only local boundary refinement, G + BR achieves a Precision of 0.668, a Recall of 0.857, and an F1-score of 0.751, almost unchanged from baseline G, while IoU increases slightly from 0.833 to 0.835, and the segment count remains 591. This result indicates that in-place boundary adjustment through local stable-point search within a single segment provides only limited improvement in boundary localization accuracy for already matched intervals and has very limited impact on overall detection performance. The fundamental reason is that the main residual error of Algorithm G is not intra-segment boundary shift, but the failure to restore long duration continuous maneuvers split by energy valleys at the candidate level. Local window search cannot operate across inter-segment gaps, so the contribution of BR depends strongly on subsequent cross-segment mechanisms. After adding cross-segment expansion and chain merging, the G + BR + GM variant shows a substantial performance leap. Recall increases from 0.857 to 0.944, F1-score and IoU rise to 0.854 and 0.898, respectively, and the total segment count converges to 558. The simultaneous increase in recall and reduction in detected segment count confirms the effectiveness of GM: it successfully reintegrates fragments incorrectly separated by low frequency energy valleys, repairing missed detections while avoiding redundant segmentation.

**Figure 17:**
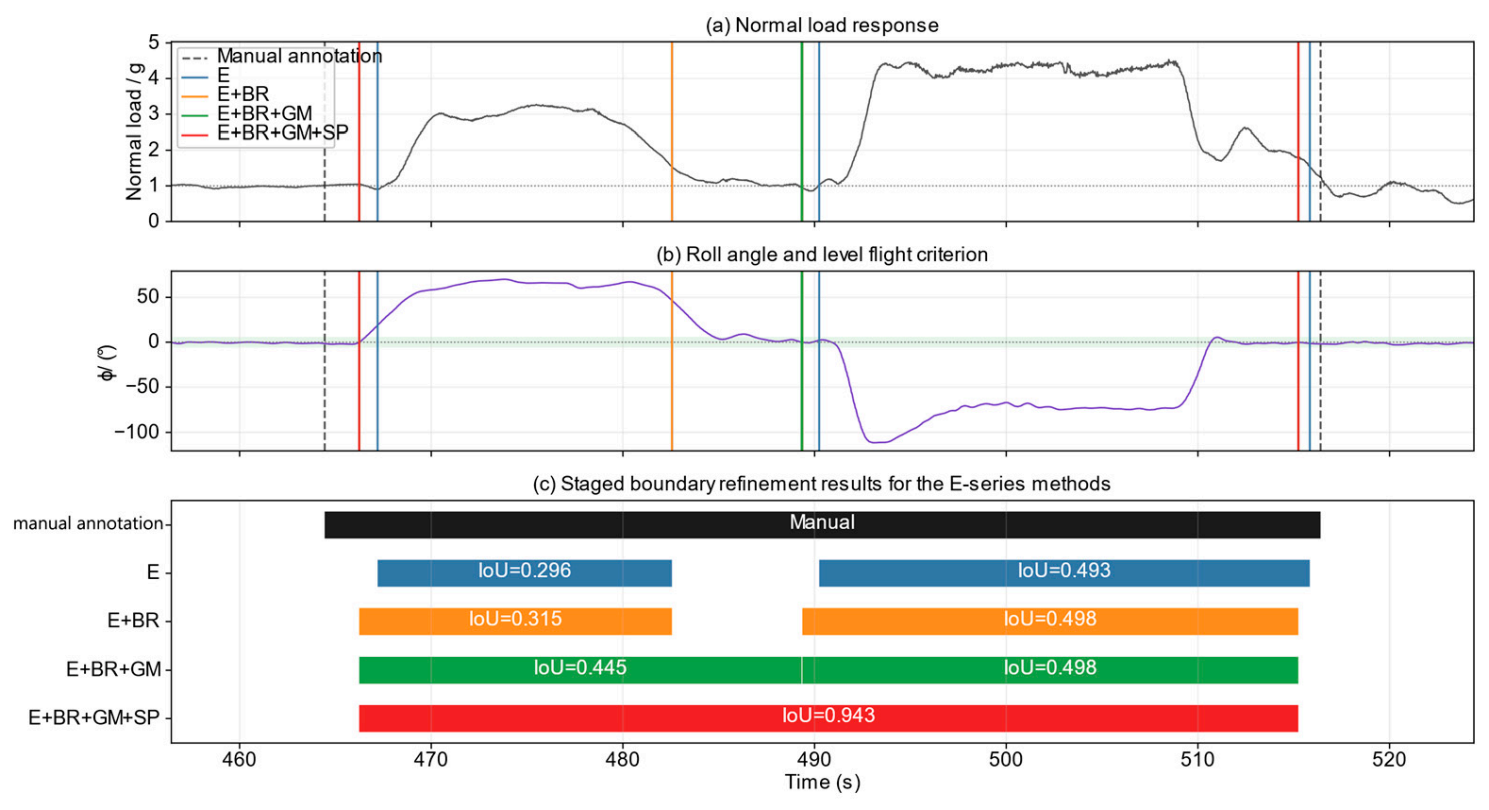
Comparison of boundary refinement effects for different ablation variants in a typical maneuver segment.

Figure 17 compares segment boundaries of the variants with manual annotations in a representative 460–520 s time window from one flight. In this interval, an instantaneous energy valley appears near 490 s, but the roll angle still maintains a large deflection, indicating internal transient fluctuation rather than maneuver recovery. A single energy criterion cannot distinguish this case, so both G and G + BR produce erroneous cuts. G + BR + GM extends the fragments through cross-segment expansion, but local gaps remain. Only at the SP stage does semantic post-processing merge the fragments completely, yielding an IoU of 0.943 and high overlap with the manual annotation. The limited increase in precision at this stage (0.780) also indicates that cross-segment merging restores maneuver integrity while introducing a small number of short noise-triggered candidate segments, providing the necessary premise for false alarm filtering in SP. After introducing SP, precision in Figure 16 increases markedly to 0.934, while F1 and IoU improve to 0.946 and 0.917, respectively, and the algorithm output count converges to 473, further reducing the deviation from the manual annotation count. Compared with previous stages, SP contributes most strongly to precision (+0.154), showing that its core value lies in fine-grained false alarm removal.

**Figure 18:**
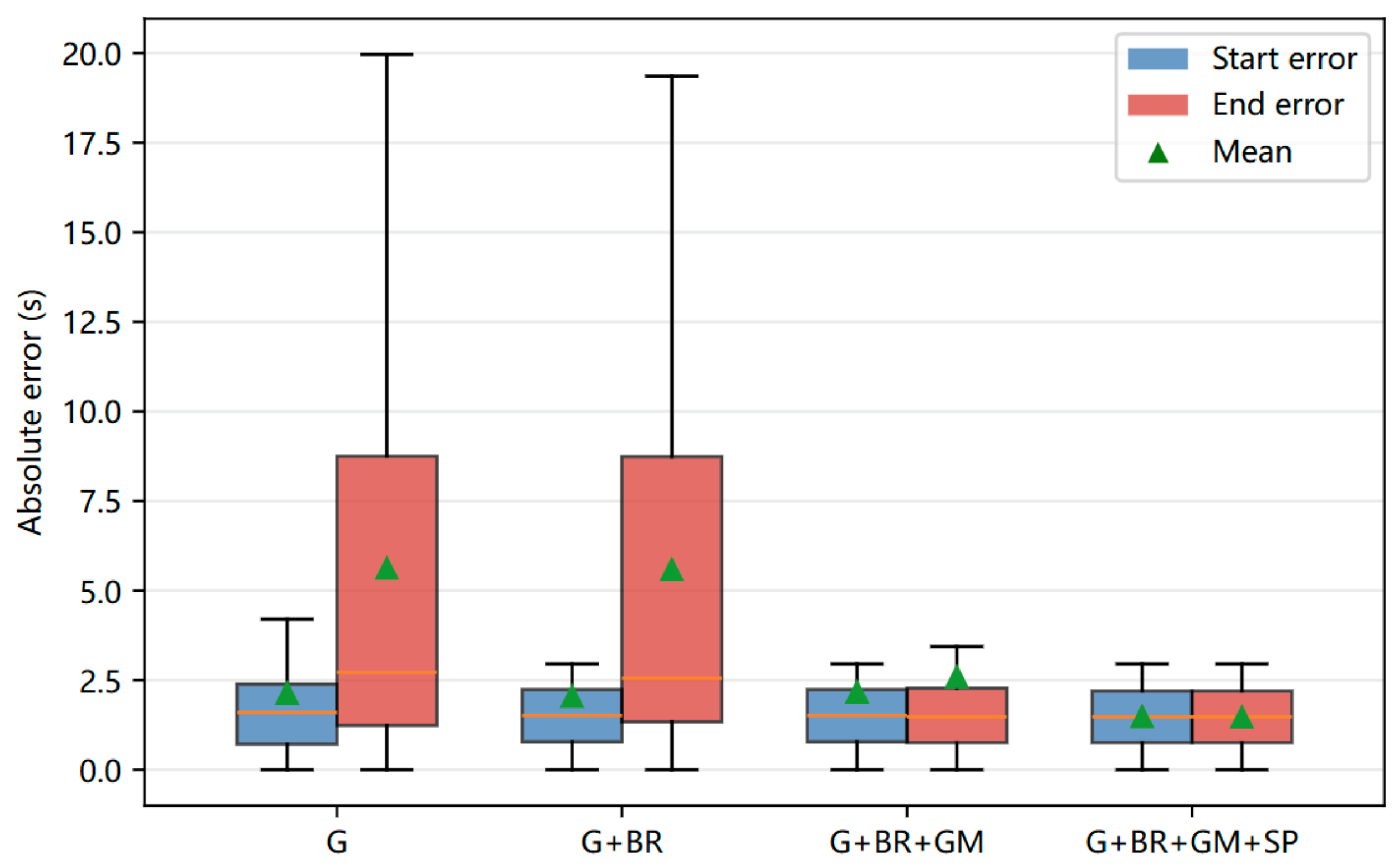
Absolute error distribution of start and end boundaries for different ablation variants.

To further quantify boundary localization accuracy, Figure 18 shows boxplots of start- and end-point absolute errors for the ablation algorithms over correctly matched intervals. In the G and G + BR stages, the upper edge of the end-point error box exceeds 8 s, and the start- and end-point distributions are clearly asymmetric, reflecting systematic boundary shrinkage caused by energy decay during maneuver recovery. With GM, the range of end-point errors narrows substantially. Ultimately, the G + BR + GM + SP variant reduces the mean absolute start- and end-point errors to 1.484 s and 1.471 s, respectively, and the error distributions on both sides become nearly symmetric. This symmetry directly reflects the role of attitude steady-state priors in boundary constraints, proving that the method has balanced detection capability for maneuver entry and recovery and effectively eliminates systematic temporal offsets caused by group delay in time frequency energy responses.

**Figure 19:**
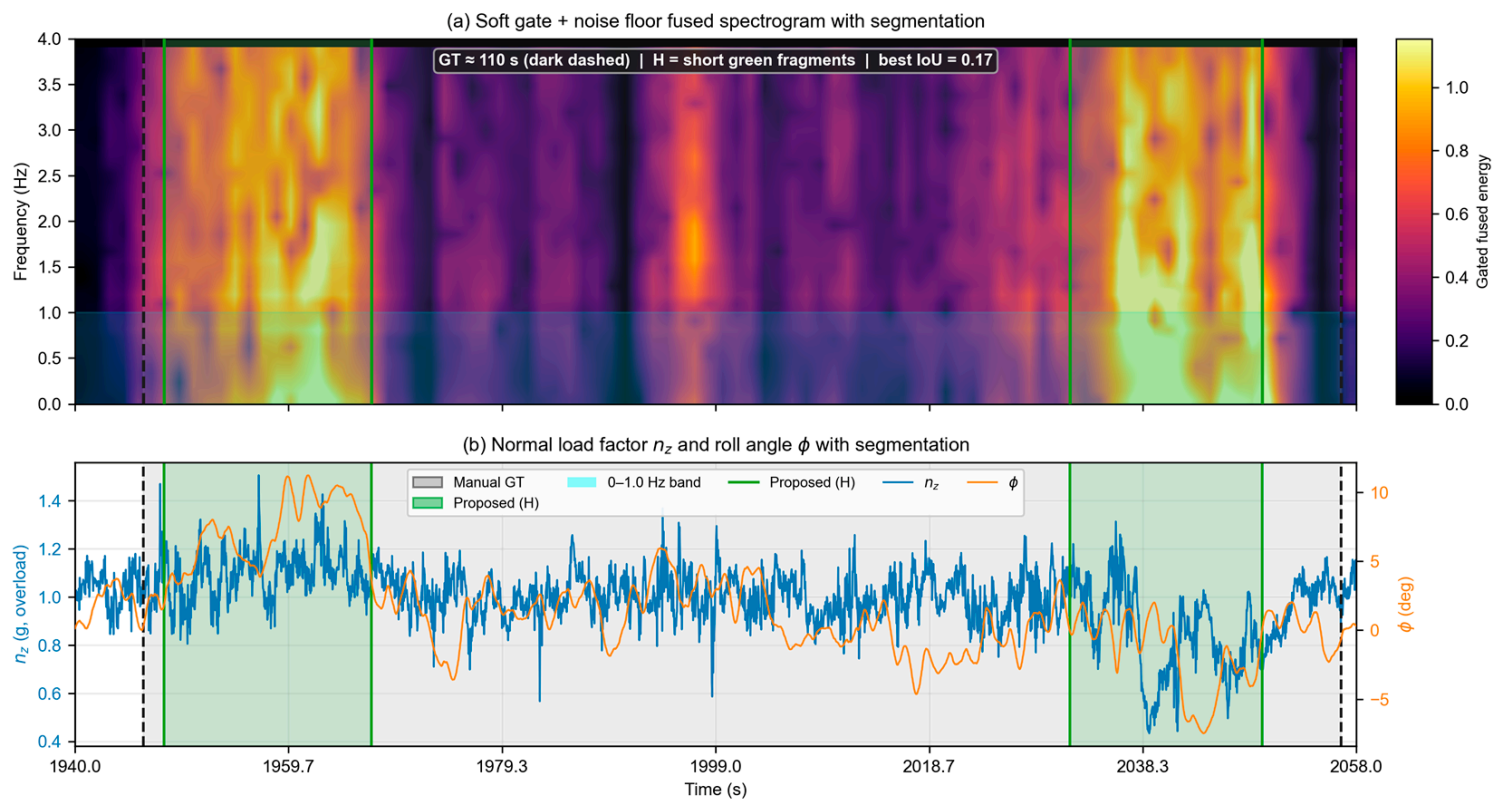
Representative under-coverage on SE_019 (1940–2058 s). (**a**) Soft gate fused spectrogram; (**b**) $n_{z}\;$ and roll ϕ. Dashed: manual GT; green: Algorithm H.

Beyond the aggregate error distributions in Figure 18, a representative residual error mode is the under-coverage of a long annotated turn (Figure 19). On SE_019 (1940–2058 s), Figure 19a shows a low frequency ridge spanning the manual ground truth interval (~110 s) on the same soft-gated plus noise floor fused spectrogram used by Algorithm H, yet H retains only short green fragments at energetic tips (best IoU = 0.17); Figure 19b shows the corresponding normal load factor $n_{z}\;$ and roll angle phi. The mid-interval energy valleys fragment the detection mask, and the resulting gaps exceed the GM merge tolerance, so the long turn is only partially recovered.

**Figure 20:**
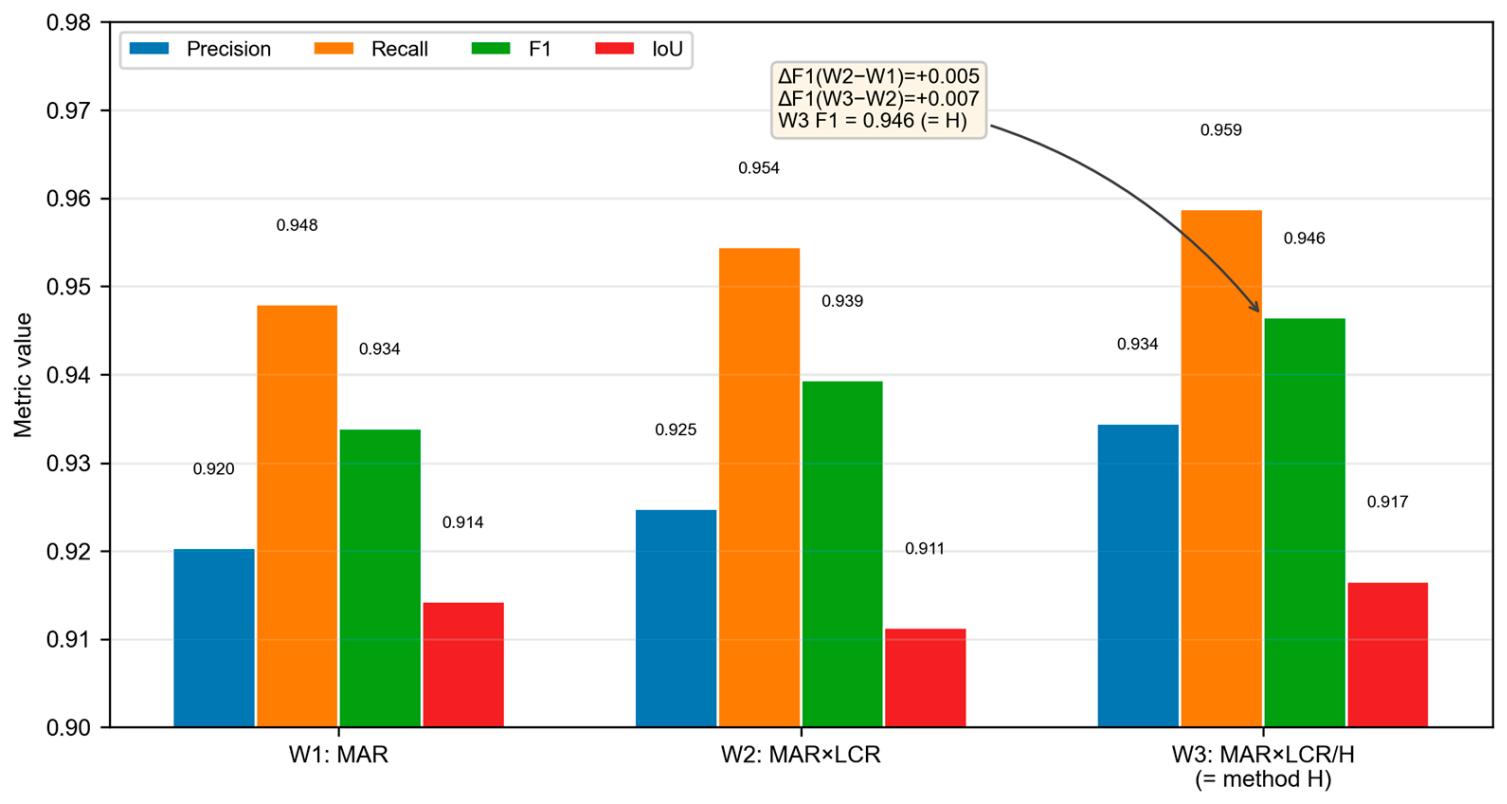
Entropy factor ablation on all 20 single-engine sorties (BR/GM/SP fixed): W1 = MAR; W2 = MAR × LCR; W3 = MAR × LCR/H.

**Figure 21:**
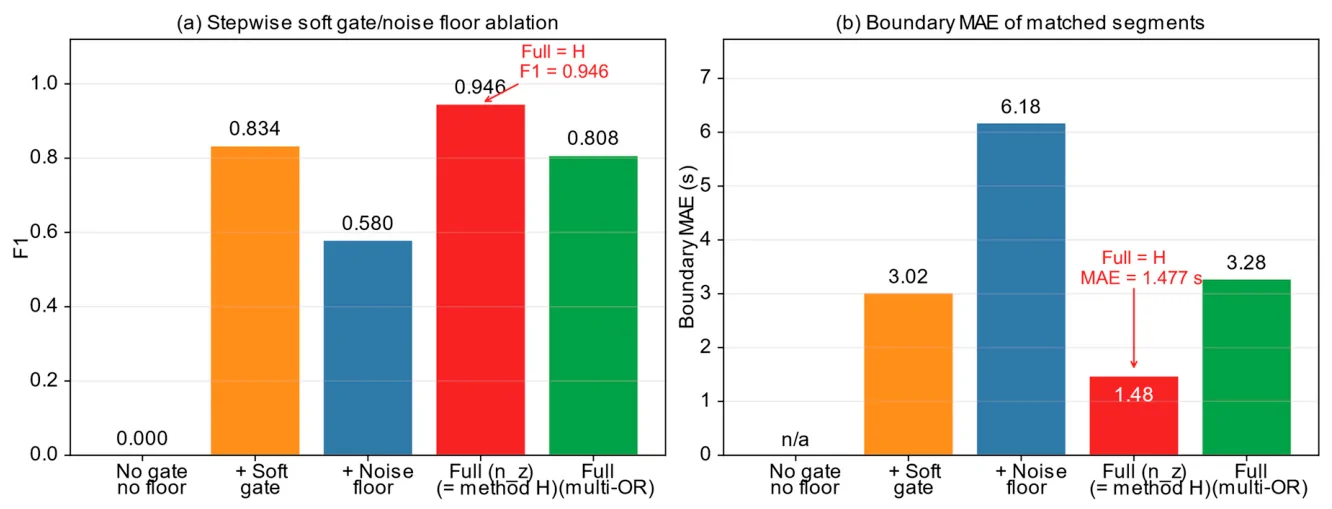
Soft gate/noise floor stepwise ablation and a multi-channel OR-gate variant (BR/GM/SP fixed). (**a**) F1; (**b**) boundary MAE (undefined when detection collapses).

The entropy factor in the channel weighting rule and the soft-gated/noise floor stages of the fusion front end are further ablated (Figure 20 and Figure 21). All runs use the same protocol and code path on all 20 single-engine sorties, with the BR/GM/SP stack held fixed; only the weighting rule or the front end variant is changed.

Entropy factor (Figure 20). From W1 (MAR; F1 = 0.934) to W2 (MAR × LCR; F1 = 0.939), F1 rises by only +0.005. Adding entropy in W3 (MAR × LCR/H; F1 = 0.946) gives a further ΔF1 = +0.007, with the largest precision gain (to 0.934) and fewer false positives (*FP*: 63 → 61 → 31). Matched IoU stays high (≈0.91–0.92) and boundary MAE is lowest under W3 (1.477 s). Under the same all-20 protocol with BR/GM/SP fixed, W3 coincides with the complete method H (Precision 0.934, Recall 0.959, F1 0.946). Entropy therefore supplies a measurable but moderate precision-leaning gain beyond MAR × LCR, rather than a cosmetic rescaling.

Soft gate and noise floor (Figure 21). With neither mechanism, detection collapses (F1 ≈ 0). Soft gate only recovers F1 = 0.834 but leaves many false positives (FP = 121; MAE = 3.02 s); noise floor only is weaker and less stable (F1 = 0.580; MAE = 6.18 s). Both restore F1 = 0.946 and MAE = 1.477 s—identical to method H on all-20—showing complementarity: the gate masks non-maneuver frames, while the noise floor cleans residual background. A multi-channel OR gate (noise floor retained) yields F1 = 0.808 (MAE = 3.28 s), below the *n_z_*-only full configuration on this load factor-rich set.

Overall, front end gating and noise handling are necessary for usable detection and, when both are enabled, recover the same all-20 operating point as method H (F1 = 0.946; MAE = 1.477 s); entropy weighting then provides a smaller but non-negligible refinement among weighting rules W1–W3—disentangling the previously confounded gap between DSP baseline D and the proposed pipeline G/H.

### 6.4. Hyperparameter Sensitivity Analysis

**Figure 22:**
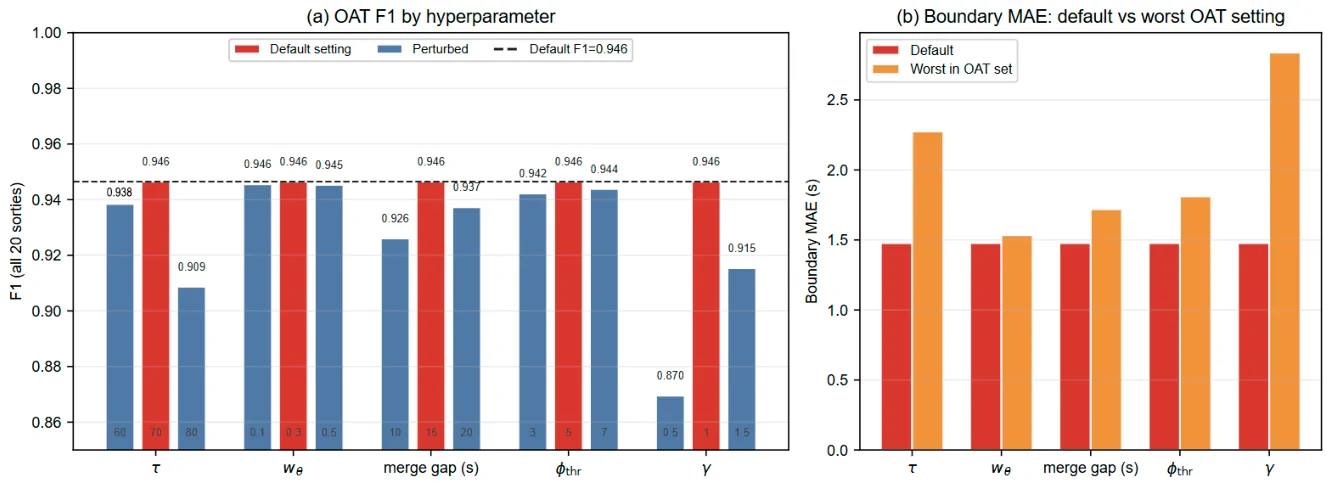
One-at-a-time hyperparameter sensitivity on all 20 single-engine sorties. (**a**) F1 by parameter (red = default); (**b**) boundary MAE of the default vs nearby settings.

Key operating parameters of the proposed pipeline are summarized in Table 6. Values were selected on the validation sorties and then frozen before test reporting. To assess robustness, a one-at-a-time (OAT) study is performed on all 20 single-engine sorties, with the remaining defaults held fixed (Figure 22). The perturbed factors are those emphasized by the reviewers and already defined in the method: the segmentation percentile τ, the pitch weight $w_{\theta }$, the SP merge gap, the roll threshold $\phi_{thr}$, and the soft gate exponent γ.

Under the default setting—identical to the complete method G + BR + GM + SP—the all-20 F1 reaches 0.946, and the matched boundary MAE is 1.477 s. Near this operating point, F1 remains high for most nearby choices. For $\tau \in \left\{60,70,80\right\}$, F1 equals 0.938/0.946/0.909; τ=70 is preferred, whereas τ=80 raises false positives and degrades both detection and localization. The pitch weight $w_{\theta }\in \left\{0.1,0.3,0.5\right\}$ is nearly insensitive (F1 0.945–0.946), so $w_{\theta }=0.3$ is retained. Shortening the SP merge gap to 10 s lowers F1 to 0.926 through excess fragmentation; extending it to 20 s yields 0.937, still below the 15 s default. The roll threshold $\phi_{thr}\in \left\{3^{\circ },5^{\circ },7^{\circ }\right\}$ likewise varies only mildly (F1 0.942–0.946), with $5^{\circ }$ best. The soft gate exponent is the most sensitive factor: γ=0.5 drops F1 to 0.870 and raises boundary MAE to 2.84 s, while γ=1.5 yields F1 0.915; γ=1.0 remains optimal. Figure 22b confirms that non-default OAT settings typically inflate boundary MAE relative to the default.

Overall, performance is stable in a neighborhood of the selected defaults, and the default combination coincides with the full proposed pipeline under the same evaluation protocol.

### 6.5. Cross-Dataset External Validation

**Figure 23:**
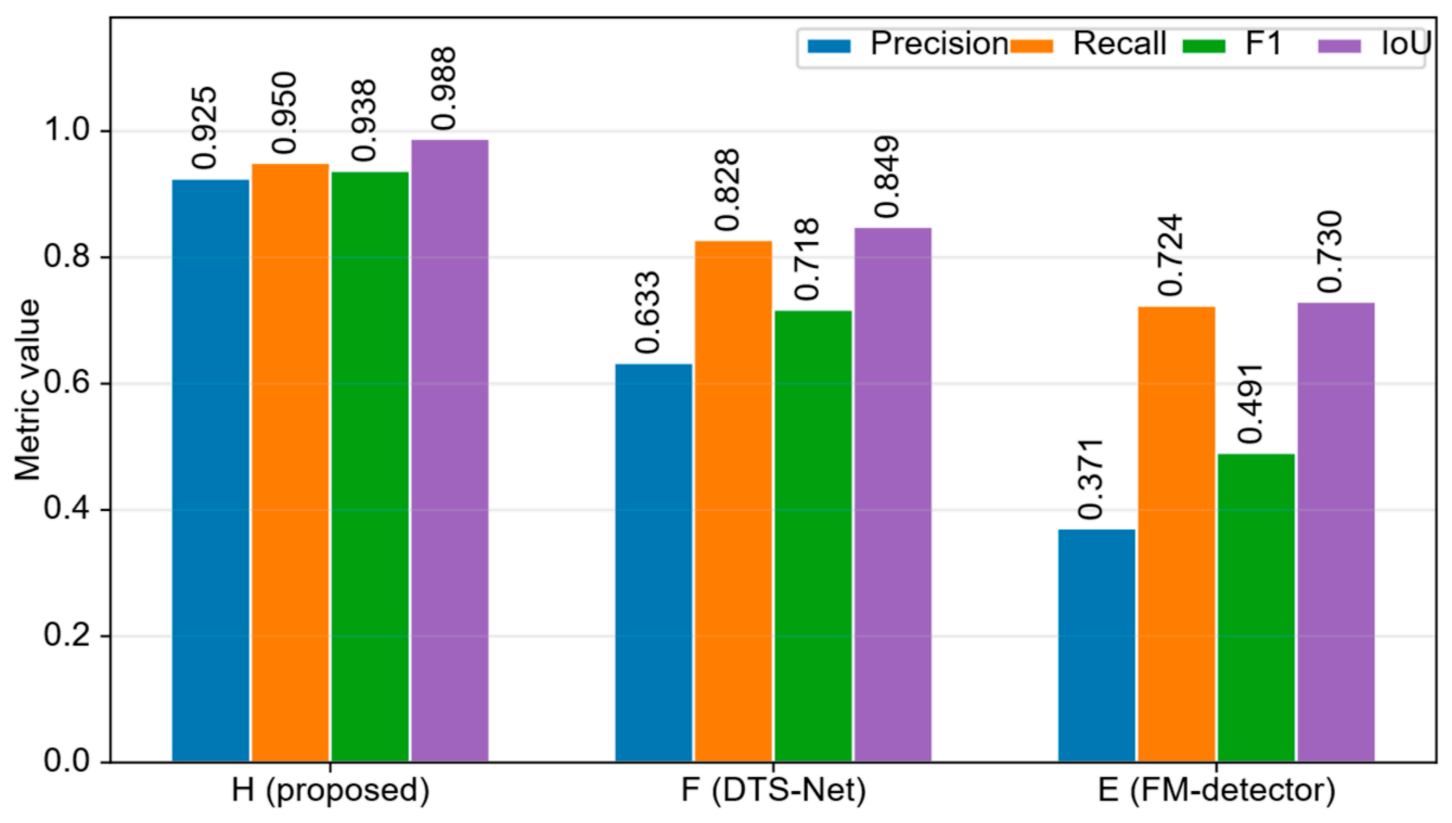
Comparison of the proposed method H with learning baselines F and E on the external dataset.

To assess whether the frozen operating point transfers beyond the development corpus, evaluation is repeated on an independent external flight data set (10 sorties) under the same IoU ≥ 0.5 matching protocol, with all thresholds left unchanged. Figure 23 compares the complete proposed method H with the learning baselines F (DTS-Net) and E (FM-detector).

On this external set, H/F/E achieve F1 = 0.938/0.718/0.491, preserving the ranking observed on the primary corpus. Method H remains clearly ahead (*p* = 0.925, R = 0.950; matched IoU = 0.988). Both learning baselines, trained only on the primary set, degrade more under the domain shift—particularly E. The result indicates that the proposed DSP pipeline retains useful detection performance on a held-out data source, whereas purely learning-based detectors are more sensitive when no target domain training data are available.

## 7. Conclusions

For flight maneuver segmentation in long duration multi-sensor records, this paper develops spectral entropy-weighted multi-channel spectrogram fusion with attitude-constrained segment structure correction. The main conclusions are as follows.

(1) Although flight parameter sampling rates and dominant bands are much lower than those of speech, maneuver processes remain non-stationary. Representing amplitude series as STFT spectrograms provides structural cues unavailable from time domain thresholds alone. With a Hann window and L=64, H=32, N=256, temporal localization and spectral discriminability are balanced for the present data.
(2) MAR, LCR, and SE quantify channel quality along temporal activity, spectral contrast, and energy distribution uncertainty. Combined with $n_{z}$ soft gating and noise floor subtraction, multi-channel fusion markedly suppresses taxiing/level flight background and low quality channels, raising F1 from 0.446 (single-channel spectrogram baseline) to 0.749. Soft gating and noise floor subtraction are jointly necessary; SE contributes a further measurable but moderate gain.
(3) The proposed adaptive flight maneuver boundary localization algorithm via spectral entropy-weighted multi-channel spectrogram fusion achieves Precision, Recall, and F1 values of 0.967 on the held-out test sorties (61 intervals). On the full primary corpus (461 intervals), used for ablation and sensitivity analyses, Precision, Recall, and F1 are 0.934, 0.959, and 0.946, with start and end boundary MAEs of 1.484 s and 1.471 s. Time frequency fusion mainly improves candidate/background separability, while BR, GM, and SP restore long-maneuver integrity. Under the same IoU protocol, the method also outperforms learning baselines and retains F1 = 0.938 on an independent external set of 10 sorties.
(4) Near the frozen defaults (τ=70%, $w_{\theta }=0.3$, merge gap = 15 s, $\phi_{thr}=5^{\circ }$, γ=1.0), performance remains stable; γ is the most sensitive factor among those tested. Broader cross-aircraft and cross-sensor evaluation under more diverse annotation protocols remains for future work.

Future work will focus on in-depth evaluation of the algorithm’s generalization and adaptive adjustment capability across aircraft types, sensor configurations, and more complex annotation protocols.

## Figures and Tables

**Table 1:** Classification of representative flight maneuver segmentation and recognition studies.

Category	Representative Studies	Task Type	Principal Limitations
Rule-based/expert systems	Wang et al. [5]; Yang et al. [6]; Zhang et al. [7]; Wang et al. [8]	Recognition only	Boundary detection reduces to single-channel threshold crossings that drift under turbulence; no correction mechanism; rules tightly coupled to specific aircraft types
Feature engineering plus probabilistic models	Zhang et al. [10]; Meng et al. [11]; Olive and Basora [12]; Lu et al. [9]	Olive and Basora [12]: boundary detection; others: recognition only	HMM boundary granularity restricted to coarse flight-phase level; temporally precise boundary localization not evaluated; others produce no boundary timestamps and are sensitive to hand-crafted priors
Data-driven neural networks	Tian et al. [14]; Wan et al. [15]; Fang et al. [16]; Zhou et al. [17]; Li et al. [18]; Xi et al. [19]; Wu et al. [20,21]; Gaugel and Reichert [22]; Bock et al. [23]	FM-detector [15], DTS-Net [20], Wu et al. [21], PrecTime [22], Bock et al. [23]: boundary detection; others: recognition only	FM-detector boundary precision limited by fixed anchor scales; DTS-Net lacks boundary supervision yielding imprecise boundaries; aviation methods require large annotated corpora; non-aviation methods need dense frame-level labels and lack aviation-specific vibration noise handling

**Table 2:** Comparison of window functions under a fixed diagnostic STFT setting: *L* = 64, *H* = 32, *N* = 256.

Window Function	Five-Channel Mean $C_{lhc}$ (dB)	Peak Sidelobe (dB, Above 4 Hz)	Main Lobe Width (Hz)
Rectangular (Boxcar)	12.80	−47.5	0.183
Hamming	13.54	−57.3	0.269
Hann	14.86	−97.2	0.293

**Table 3:** Maneuver category distribution across the datasets.

Maneuver Category	Primary Corpus		External Validation Set	
	(Single-Engine, 20 Sorties)		(Twin-Engine, 10 Sorties)	
	Intervals	Proportion (%)	Intervals	Proportion (%)
Loop	58	12.6	27	12.2
Half Cuban Eight	54	11.7	21	9.5
Horizontal Figure Eight	57	12.4	19	8.6
High Speed Roll	79	17.1	44	19.9
Turn	86	18.7	56	25.3
Circling	53	11.5	23	10.4
Combined Maneuver	74	16.1	31	14
Total	461	100	221	100

**Table 4:** Comparison of flight maneuver segmentation algorithms.

ID	Algorithm Name	Algorithm Characteristics
A [5]	$n_{z}\;$ Fixed threshold method	Single-channel $n_{z}\;$ time domain amplitude; adaptive percentile threshold; no boundary correction; lowest computational cost
B	Multi-channel time domain energy fusion	$n_{z}\;$ + p + r three-channel normalized energy weighting; time domain processing; adaptive percentile threshold; no frequency selectivity
C	$n_{z}\;$ Short time variance adaptive threshold method	Single-channel $n_{z}\;$ local variance; adaptive percentile threshold; sensitive to transient disturbances but insufficient for gradual maneuvers
D	$n_{z}\;$ Spectrogram method	Single-channel $n_{z}\;$ STFT time-frequency energy; dual adaptive thresholds for low frequency detection and high frequency rejection; single-channel information source
E [15]	FM-detector-style segmenter	End-to-end flight-maneuver detector-style baseline trained on single-engine fighter train/val sorties only; same IoU protocol at test time
F [20]	Deep temporal segmentation network (DTS-Net)	Literature-style deep temporal segmentation trained on single-engine fighter train/val sorties only; same IoU protocol at test time
G	Multi-channel entropy-weighted spectrogram fusion	Five-channel MAR-LCR spectral entropy-weighted fusion with soft gating and noise floor subtraction; no attitude correction (formerly Algorithm E)
H	Proposed complete method (G + BR + GM + SP)	Algorithm G plus trend-aware local boundary refinement (BR), cross-segment expansion/chain merging (GM), and semantic post-processing (SP)

**Table 5:** Staged ablation methods for the fused spectrogram segmentation algorithm.

Method	Added Module	Main Technical Characteristics
G	-	Five-channel MAR-LCR spectral entropy-weighted fused spectrogram; adaptive percentile threshold for candidate detection; no boundary correction.
G + BR	Local boundary refinement (BR)	Searches for the optimal stable point within a ±1.25 s local window around each candidate endpoint using trend-aware roll and pitch scores; performs in-place single-segment boundary adjustment.
G + BR + GM	Cross-segment expansion and chain merging (GM)	Performs chain merging for adjacent segments with gaps ≤ 15 s and roll angle continuously exceeding 5 degrees during the interval; maximum chain length is three segments; restores long duration continuous maneuvers split by energy valleys.
G + BR + GM + SP	Semantic post-processing (SP)	Adds semantic merging of same-direction continuous roll actions, false alarm filtering for overly long low attitude amplitude segments, and removal of fragments shorter than 1 s.

**Table 6:** Key operating parameters of the proposed method.

Parameter	Value	Role
*L*/H/N	64/32/256	STFT window/hop/DFT size (Hann; $f_{s}=25$ Hz)
$k_{0}$	>0.20 for ≥5 frames	Flight start frame from soft gate
γ	1.0	Soft gate exponent
τ (%)	70	Adaptive segmentation percentile
$T_{min}$/$T_{gap}$ (s)	3.0/3.8	Minimum duration/max intra-mask gap
$B_{d}$/$B_{e}$	2/1	Morphological dilate/erode (frames)
BR refine window (s)	±1.25	Local boundary refinement search window
$w_{\theta }$	0.3	Trend-aware pitch weight in BR
$\phi_{thr}$ (°)	5	Level flight roll threshold in BR
SP merge gap (s)	15	Chain-merge gap for adjacent segments (SP)
SP max chain length	3	Maximum segments merged in one SP chain
SP short fragment cut (s)	<1	Discard residual fragments shorter than 1 s

## Data Availability

The data presented in this study are available on request from the corresponding authors due to privacy.
